# A novel interaction fingerprint derived from per atom score contributions: exhaustive evaluation of interaction fingerprint performance in docking based virtual screening

**DOI:** 10.1186/s13321-018-0264-0

**Published:** 2018-03-16

**Authors:** Julia B. Jasper, Lina Humbeck, Tobias Brinkjost, Oliver Koch

**Affiliations:** 1 Faculty of Chemistry and Chemical Biology, TU Dortmund University, Otto-Hahn-Str. 6, 44227 Dortmund, Germany; 2 Department of Computer Science, TU Dortmund University, Otto-Hahn-Str. 14, 44227 Dortmund, Germany

**Keywords:** Interaction fingerprints, Docking, Protein ligand interactions, Scoring, Pose prediction, Virtual screening, Scaffold hopping

## Abstract

Protein ligand interaction fingerprints are a powerful approach for the analysis and assessment of docking poses to improve docking performance in virtual screening. In this study, a novel interaction fingerprint approach (PADIF, protein per atom score contributions derived interaction fingerprint) is presented which was specifically designed for utilising the GOLD scoring functions’ atom contributions together with a specific scoring scheme. This allows the incorporation of known protein–ligand complex structures for a target-specific scoring. Unlike many other methods, this approach uses weighting factors reflecting the relative frequency of a specific interaction in the references and penalizes destabilizing interactions. In addition, and for the first time, an exhaustive validation study was performed that assesses the performance of PADIF and two other interaction fingerprints in virtual screening. Here, PADIF shows superior results, and some rules of thumb for a successful use of interaction fingerprints could be identified.

**Electronic supplementary material:**

The online version of this article (10.1186/s13321-018-0264-0) contains supplementary material, which is available to authorized users.

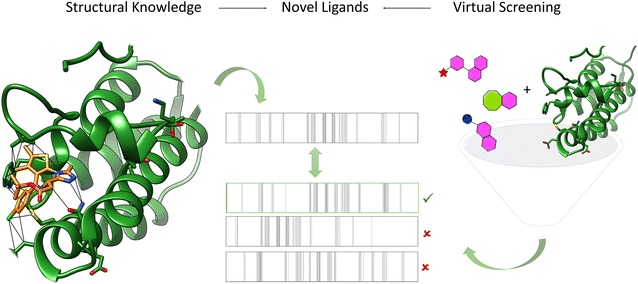

## Background

Docking based virtual screening and molecular design have become an important part of the structure based drug discovery and design process [1]. A major challenge of these approaches is the correct assessment of the resulting docking poses to identify the most likely binding mode for each molecule and generate a relative ranking of different molecules [2]. Due to the serious difficulties of this task, many different scoring functions have been developed which are classically categorised into force field, empirical and knowledge based approaches [3, 4]. However, they were designed to be universally applicable for every protein. It is therefore reasonable to also incorporate available structural data about a specific target protein for creating a tailor-made scoring especially fitted for this one protein of interest. Interaction fingerprints present an efficient way to achieve this by assessing docking poses of potential new ligands via a simple comparison to the respective interactions of known protein–ligand complex structures. This interaction based comparison is independent of the molecular structure and thus a promising tool for identifying new ligands with similar interactions but completely different core structures. This so called “scaffold hopping”, originally devised by Schneider et al., is an important task in medicinal chemistry [5, 6]. Interestingly, a former study suggests that interaction fingerprint based scoring outperforms conventional scoring functions with respect to scaffold hopping enrichment [7]. In the past, various interaction fingerprints have been developed and successfully employed for post processing of docking poses. For a detailed overview and description of methods, the interested reader may refer to literature such as [8].

One of the first and well-established fingerprints is the structural interaction fingerprint (SIFt) [9]. It is a binary fingerprint build up from a seven bit vector for each amino acid that encodes if an interaction with the ligand occurs, whether main chain and/or side chain atoms are involved, whether there is a polar or nonpolar interaction and whether the residue provides a hydrogen bond donor or acceptor [9]. SIFt and several extensions have successfully been used for various tasks: profile-SIFts (p-SIFts) were applied for the enrichment of kinase inhibitors and for analysing their selectivity [10]. Weighted SIFts (w-SIFts) incorporate knowledge about ligand activities and were used to rank compounds of a target by their potency [11]. IFP, another modified implementation of SIFt, was successfully used for improving fragment and scaffold docking [12]. It does not distinguish between main-chain and side-chain atoms but incorporates more interaction types than the original SIFt [12]. Besides, the concept was extended to atom based fingerprints with further modifications: An expanded interaction fingerprint approach incorporates hydrogen-bonding strength and/or accessibility of the hydrogen bonding groups as well as geometric arrangement [13]. CHIF, the knowledge-based interaction fingerprint scoring by Mpamhanga et al. [14], combines similarity coefficients with the scores of Goldscore to yield a binding knowledge modified score [14]. The authors also introduce a multiple reference scoring scheme by creating a frequency-weighted fingerprint from many reference structures. For the Bissantz ER-receptor test set, the applied scoring schemes yielded significant improvement compared to Goldscore, and multiple reference scoring seemed to outperform the single reference scoring [14].

Besides, more complex fingerprint concepts were developed that often rely on encoding relative positions or distances of interacting atoms or pharmacophore features. The structural protein ligand interaction fingerprint (SPLIF) stores interactions implicitly with help of extended connectivity fingerprints [15]. The atom-pairs-based interaction fingerprint (APIF) encodes relative positions of pairs of interacting atoms in a 294 bit fingerprint [16]. Based on the work of Mpamhanga et al., the authors also employed a combined score with Goldscore and improved the enrichment compared to Goldscore alone [14, 16]. TIFP utilises so called interaction pseudoatoms which are defined based on the pharmacophoric types of interacting protein and ligand atoms [17]. The advantage of such residue independent methods is that they are not binding site specific. Thus, fingerprints from complex structures of different proteins can be compared.

The described methods and their applications give reasons to hope that interaction fingerprints can aid the analysis of docking results. However, most approaches were only validated on selected examples. This makes the assessment of their general applicability and also the comparison of different fingerprint methods difficult. In this study, we therefore provide an in-depth analysis of the performance of our newly developed protein per atom score contributions derived interaction fingerprint (PADIF) as well as of two other interaction fingerprints in docking based virtual screening on the well-established directory of useful decoys (DUD) [18]. As representatives for existing fingerprint methods, we chose IFP and TIFP to incorporate both a simple, residue based interaction fingerprint, and a more complex, binding site independent approach. Both are implemented in the tool IChem which is available upon request [12, 17]. So, an exhaustive analysis of interaction fingerprint performance in virtual screening is presented that can aid the user to decide when and how these methods can best be employed.

## Results and discussion

### PADIF scoring

The PADIF approach consists of the interaction fingerprint and a specific scoring scheme which was specifically designed to combine the strengths of fingerprint methods with conventional scoring functions. The fingerprint is atom based since this allows for an exact and easily interpretable analysis of docking poses. For improving the information content compared to most SIFt related fingerprints, PADIF incorporates the strengths of the different interactions as well as the presence of unfavourable interactions. This is achieved by exploiting the per atom score contributions of the protein atoms which are calculated for each pose during docking with GOLD [19]. These contributions are binding site specific, atom based, quantitative, differentiate between favourable and unfavourable interactions via prefix and can be easily extracted from the docking pose output files. Although PADIFs were only made up from GOLD scoring function contributions in the present study, the underlying procedure should in principle be applicable to any other scoring function that allows to output atom specific contributions.

With respect to the scoring, we wanted to incorporate the lessons learned from studies such as [14], e.g. usage of multiple reference scoring coupled with frequency based weighting of the interactions. This does not only prevent that the resulting similarity score is strongly biased towards a single reference ligand but also yields information about more and less frequently occurring interactions. It has to be noted that the frequency of an interaction does not necessarily correlate with the importance of this interaction. However, as a starting point and especially if complexes with diverse ligands are available, the weighting according to frequency is reasonable to extract as much information as possible. Furthermore, our approach discriminates between favourable and unfavourable interactions and penalizes the latter, which is not possible with conventional, binary interaction fingerprints. Thus, rather than employing a Tanimoto coefficient or a Euclidean distance, our PADIF similarity score is basically constructed by counting matching favourable interactions weighted by frequency and decreasing the score upon unfavourable interactions again weighted by frequency.

In addition, we tried to address a problem that is known for conventional scoring [20]: By summing up matching interactions, smaller molecules will inevitably get lower similarity scores than larger ones despite a very good matching. In order to relatively increase the score of poses of small, yet nicely matching molecules, an overlap factor that considers the relative matching was introduced. Thus, a fingerprint which shows only a low number of favourable interactions that all match with those in the combined and larger reference fingerprint has a perfect overlap factor. On the other hand, the similarity score of a fingerprint with a high number of favourable interactions that only partly match to the reference gets decreased. This procedure aims to prevent the enrichment of very large molecules.

For conventional scoring (and thus for PADIF generation), the default scoring function of GOLD, ChemPLP [21], was used since it yielded the overall best results in former benchmarking studies [22, 23]. However, the approach could likewise be used with all GOLD scoring functions. In order to combine fingerprint similarity and conventional score, a joint score from PADIF similarity and ChemPLP score is additionally introduced. The presented approach and study thus aim to take up the lessons learned from previous publications and the strengths of former fingerprint approaches and to combine it with a non-binary fingerprint that integrates favourable and non-favourable interactions.

### Pose recovery

The identification of a ligand’s most likely binding mode is one of the major tasks of a scoring function [2]. Ideally, poses with a low root mean square deviation (RMSD) to the native binding mode of the query ligand should be placed on top of the ranking. In order to evaluate the performance of PADIF in pose recovery, 100 diverse docking poses (inter pose RMSD > 1.5 Å) per ligand were generated for a suitable subset of 61 complexes of the Astex diverse dataset [24] and then ranked by ChemPLP and PADIF score. RMSD values below 2.0 or 2.5 Å are often suggested as thresholds for a “good” pose [25–27]. In the present study, pose recovery works well for ChemPLP and PADIF scoring: For both, 80% of the top ranked poses have an RMSD below 2.5 Å. The 2.0 Å threshold is achieved for 74 and 77% of the top ranked poses from PADIF and ChemPLP scoring, respectively, a 1.0 Å threshold for 49 and 51%. This implies that both methods are well suited for positioning ligands. A more sophisticated PADIF scoring scheme that was specifically developed for pose recovery yielded slightly better results (see Additional file 1: S1).

It is noteworthy that the reference complexes for PADIF based scoring were selected in a way that the respective molecules were not too similar to the docked compound to avoid bias and to show the method’s broad applicability. However, when the user wants to find the most likely binding mode of a molecule of interest, it would of course be sensible to specifically select the most suitable reference complexes for this task. Besides, it is possible that the full potential of PADIF scoring could not be exploited due to a simplification in the preparation of the reference fingerprints: Because of the high number of reference complexes, they were not prepared and rescored individually, but all complexes of one protein were superposed and all reference ligands were rescored in the structure that was used for docking. This might introduce a bias even for very good superposition of proteins since slight changes of the distance between protein and ligand atoms may result in huge changes in the ChemPLP scores. Thus, the values for the reference fingerprints might be falsified.

Altogether though, the analysis confirms that both ChemPLP and PADIF scoring are capable of identifying the right binding mode of a given ligand, which is the precondition for their use in virtual screening. This is in agreement with already presented studies which showed that pose prediction ranking by interaction fingerprint similarity usually performs better than or equally to the conventional scoring functions [13, 16, 17].

### Virtual screening

**Fig. 1:**
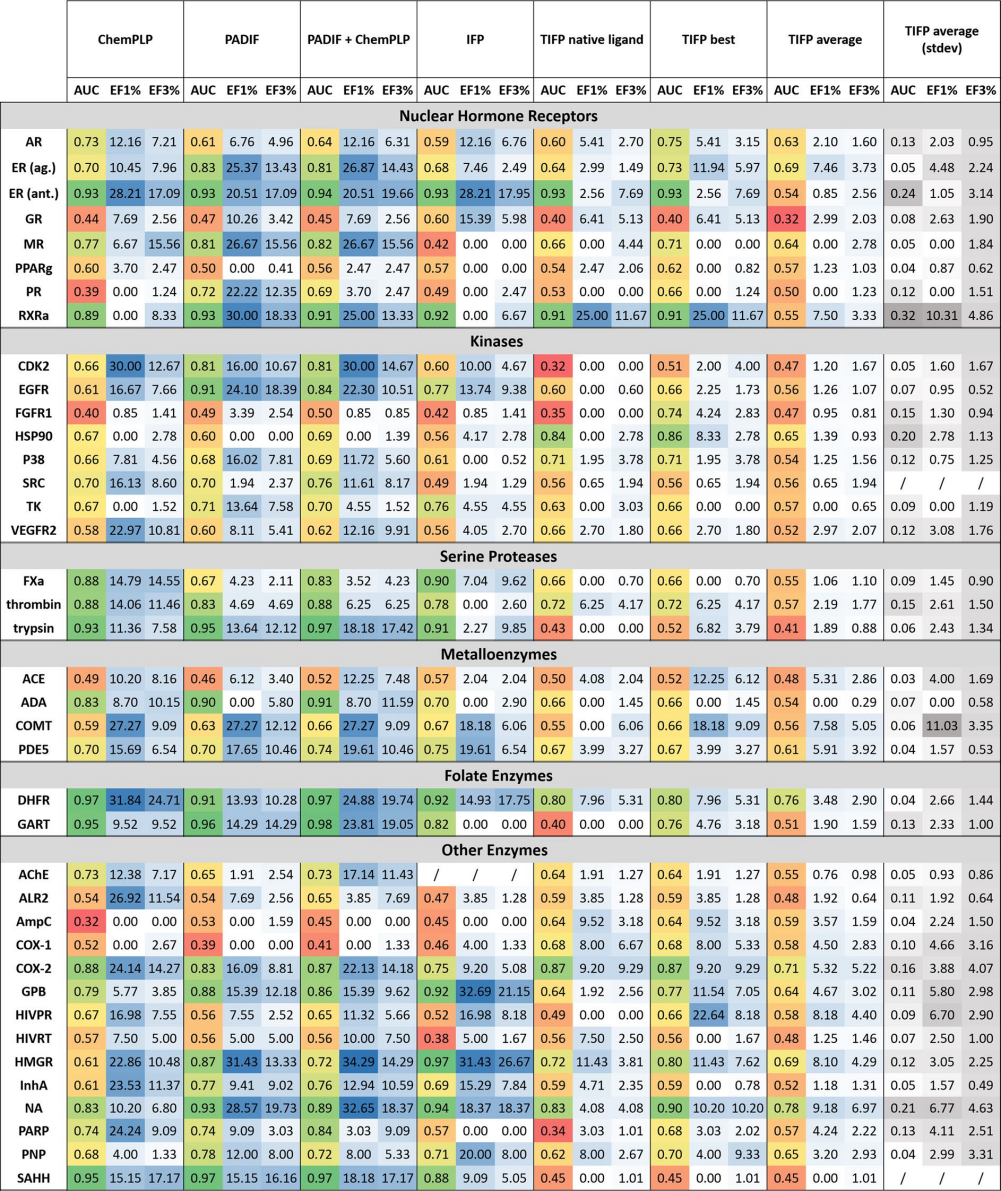
Virtual screening performance. AUC and EFs for ChemPLP, PADIF, a combination of PADIF and ChemPLP, IFP and TIFP are presented. For PADIF, a multiple reference scoring was employed (reference complexes in Additional file 1: S3). For IFP, one single reference scoring was carried out using only the complex of the structure used for docking as a reference (Additional file 1: S3). In case of TIFP, several single reference scorings were done with all reference complexes (the same as used for PADIF and IFP), resulting in one ranking for each reference complex. Therefore, multiple results are shown for TIFP: native = ranking based on the same reference complex as for IFP; best = ranking based on the reference complex that gave the best results (AUC based); average: averaged values of all individual rankings. For AChE, no ranking based on IFP could be generated since the tool yielded an error when processing the docking poses. AUC values are coloured from dark green (perfect AUC of 1.0) to dark red (random enrichment); EFs are coloured dependant on their values (from white to dark blue)

The relative ranking of poses of different ligands is a more challenging task than binding mode prediction as the molecules might differ in size and chemical properties. In order to assess the performance of different fingerprint approaches (PADIF, IFP, TIFP), conventional ChemPLP scoring and a combination of PADIF and ChemPLP in virtual screening, docking experiments were performed for 39 datasets of the DUD [18], comprising targets from different, highly relevant protein classes. As measure for virtual screening success, the overall enrichment in form of the AUC (area under the receiver operating characteristic (ROC) curve) and the early enrichment in form of EF_1%_ and EF_3%_ (enrichment factor) were assessed. Figure 1 shows the results for the different approaches.

For conventional scoring with ChemPLP, the obtained results for the AUC values are in high accordance with a previous comparative docking experiment of the GOLD scoring functions on the DUD datasets [23]. With respect to the different protein classes, it is apparent that ChemPLP performs exceptionally well for folate enzymes (average AUC of 0.96) and serine proteases (average AUC of 0.90). For nuclear hormone receptors, metalloenzymes and other enzymes the results strongly depend on the protein, whereas the overall performance for kinases (average AUC of 0.62) is worst. This trend was also observed when the virtual screening accuracy of other docking programs were compared using DUD [26]. On average ChemPLP exhibits an AUC of 0.69 over all 39 datasets.

By employing PADIF scoring or a combination of PADIF with ChemPLP, the average AUC can be increased to 0.73 and 0.74, respectively. Especially for kinases, the incorporation of structural knowledge leads to an increased overall AUC (0.69 and 0.7 compared to 0.62). In total, PADIF based scoring can increase the AUC compared to ChemPLP by at least 0.1 in nine cases; for four of these proteins (progesterone receptor (PR), epidermal growth factor receptor (EGFR), AmpC β-lactamase (AmpC), HMG-CoA reductase (HMGR)), the AUC is even improved by ≥ 0.2. An AUC decrease of at least 0.1 compared to ChemPLP is only observed for four proteins (once ≥ 0.2). For the combination of PADIF and ChemPLP, an improvement of ≥ 0.1 is also observed nine times (twice ≥ 0.2), and only in one case [cox1-containing prostaglandin H(2) synthase-1 (COX-1)], the resulting AUC is decreased by ≥ 0.1 compared to ChemPLP scoring. With respect to the early enrichment, all three methods yield EF_1%_ and EF_3%_ values significantly larger than 1 for the majority of complexes, with excellent early enrichment for estrogen receptor (ER) (antagonist), cyclin dependant kinase II (CDK2), EGFR, catechol O-methyl-transferase (COMT), dihydrofolate reductase (DHFR), HMGR, and S-adenosyl-homocysteine hydrolase (SAHH). Particularly interesting is that PADIF achieves a very good early enrichment in some cases for which ChemPLP shows low EFs [PR, retinoid X receptor (RXR), thymidine kinase (TK), purine nucleoside phosphorylase (PNP)], even when used in combination with ChemPLP. These results illustrate that the structural knowledge incorporated in the PADIF score can compensate potential deficiencies of the conventional scoring function. This effect is most pronounced for the datasets of EGFR, HMGR and especially PR.

With an average AUC of 0.68, IFP similarity scoring exhibits an overall performance comparable to ChemPLP, although the ranking is merely based on the Tanimoto coefficient of the comparison to only one single reference complex per protein. Like ChemPLP, it performs exceptionally well for the folate enzymes and serine proteases (average AUC = 0.87 and 0.86). For some cases in which ChemPLP yields almost perfect rankings [thrombin, adenosine deaminase (ADA), glycinamide ribonucleotide transformylase (GART), cyclooxygenase 2 (COX-2)], the AUC of IFP scoring is reduced but still very good. An improvement in AUC of ≥ 0.1 compared to ChemPLP is achieved for glucocorticoid receptor (GR), PR, EGFR, AmpC, glycogen phosphorylase b (GPB), HMGR and neuraminidase (NA), with a massive increase of ≥ 0.3 for HMGR. For GR, IFP is the only method that achieves both a reasonable overall enrichment (0.6) and also a very good early enrichment. However, the results for the general early enrichment show that often the EF_1%_ and EF_3%_ values are lower than for ChemPLP or the PADIF approaches. This might be owing to the fact that only one reference complex was used which likely does not perfectly represent the interactions of all actives in the dataset, so that molecules with additional interactions will appear later in the ranking. Altogether though, the results of the IFP approach (which was originally introduced to optimize fragment and scaffold docking [12]) demonstrate that a very simple similarity measure can work for differentiating actives from decoys, although the performance varies between different protein classes. IFP exhibits very good AUC values ≥ 0.9 for several proteins, but also reduced AUC values in comparison to ChemPLP for other proteins.

For the binding site independent TIFP, the Tanimoto similarity was not only calculated for the complex of the protein used for docking but individually for all reference complexes that were also used by PADIF scoring, resulting in multiple similarity based rankings for each dataset. Figure 1 contains both the results of the best ranking selected based on AUC and the averaged results with standard deviations. In addition, the results based on the same reference complex as used for IFP are shown. When considering only the best ranking per dataset, TIFP obtains an average AUC value of 0.68 which is similar to IFP and ChemPLP, and shows massive improvement for PR, fibroblast growth factor receptor 1 (FGFR1), heat shock protein 90 (HSP90), AmpC, COX-1 and HMGR compared to ChemPLP. For FGFR1, AmpC and COX-1 it is the only method that achieves a satisfactory overall and early enrichment clearly distinct from chance. When directly comparing the performance of IFP and TIFP with the same reference complex, it is quite surprising that the performance of both methods is rather different for many datasets: While IFP yields a reasonable enrichment for GR but fails for MR, the results from TIFP are vice versa. A massive discrepancy can also be observed for CDK2 (IFP AUC = 0.60, TIFP AUC = 0.32), GART (IFP AUC = 0.82, TIFP AUC = 0.40), GPB (IFP AUC = 0.92, TIFP AUC = 0.64), poly (ADP-ribose) polymerase (PARP) (IFP AUC = 0.57, TIFP AUC = 0.34) and SAHH (IFP AUC = 0.88, TIFP AUC = 0.45) as well as for HIV-1 reverse transcriptase (HIVRT) (IFP AUC = 0.38, TIFP AUC = 0.56), COX-1 (IFP AUC = 0.46, TIFP AUC = 0.68), and aldose reductase (ALR2) (IFP AUC = 0.47, TIFP AUC = 0.59). These findings demonstrate that, even with the same structural input, different fingerprint methods can yield massively different results. This implies that the user should do some tests first to find the most suited fingerprint for his task, just like it is common practice for conventional scoring functions.

With respect to the choice of one or more reference complexes, the high standard deviations for the average TIFP performance for several datasets suggest that the selection of a suitable structure is crucial for scoring success. Although one could think of some reasonable rules of thumb here (for example simply taking the complex with the ligand with the highest affinity), our results propose that the differences in scoring performance are not always easily explainable. For example, complexes with the ligands N-trifluoroacetyl-beta-D-glucopyranosylamine and *N*-acetyl-beta-d-glucopyranosylamine were used as references for GPB. The two molecules are highly similar in structure and have comparable affinities in the micromolar range [28]. However, with 0.48 and 0.76, the corresponding TIFP AUC values differ massively. Such discrepancies will be difficult to predict prospectively and advocate the usage of a multiple reference scoring like in the PADIF approach. By combining an arbitrary number of references into a merged fingerprint and weighting the interactions by frequency, one does not only circumvent the difficult decision for the best suited reference but can incorporate the knowledge stored in several structures. Besides, due to the combination and weighting process, the approach is very robust, so that likely even the consideration of one or two less suited reference structures can be compensated. This is supported by the good performance of the PADIF approach compared to the averaged results of TIFP.

However, when assessing the performance of TIFP, one has to keep in mind that it was not designed for scoring of virtual screening results but rather as a universal method to convert coordinates of protein and ligand atoms and their pharmacophoric properties into a simple fingerprint independent of residue numbers and absolutes coordinates. The authors employed it for analysing the relation between interaction pattern similarity and ligand or binding site similarity among thousands of complexes of diverse proteins [17]. This abstraction from specific residues is an enormous advantage for addressing questions involving multiple different proteins but might be disadvantageous for scoring in a traditional virtual screening scenario with only one protein. Furthermore, like for IFP, the smaller version of TIFP was used and it is possible that the unpruned one would yield improved results.

Altogether, the results suggest that interaction fingerprint similarity is indeed a suitable tool for ranking poses in a docking based virtual screening. All methods tested here lead to a reasonable enrichment for most proteins. Comparison of the performance however implies that, for classical virtual screening, a residue or atom based interaction fingerprint is more suited since this task demands exactness rather than fuzziness and universal applicability. Besides, the good performance of the PADIF approaches demonstrates that (1) it is legitimate and useful to exploit the per atom score contributions of GOLD scoring functions for building interaction fingerprints and (2) that the employed multiple reference scoring combined with frequency based weighting seems to be a robust and promising way for ranking poses.

**Fig. 2:**
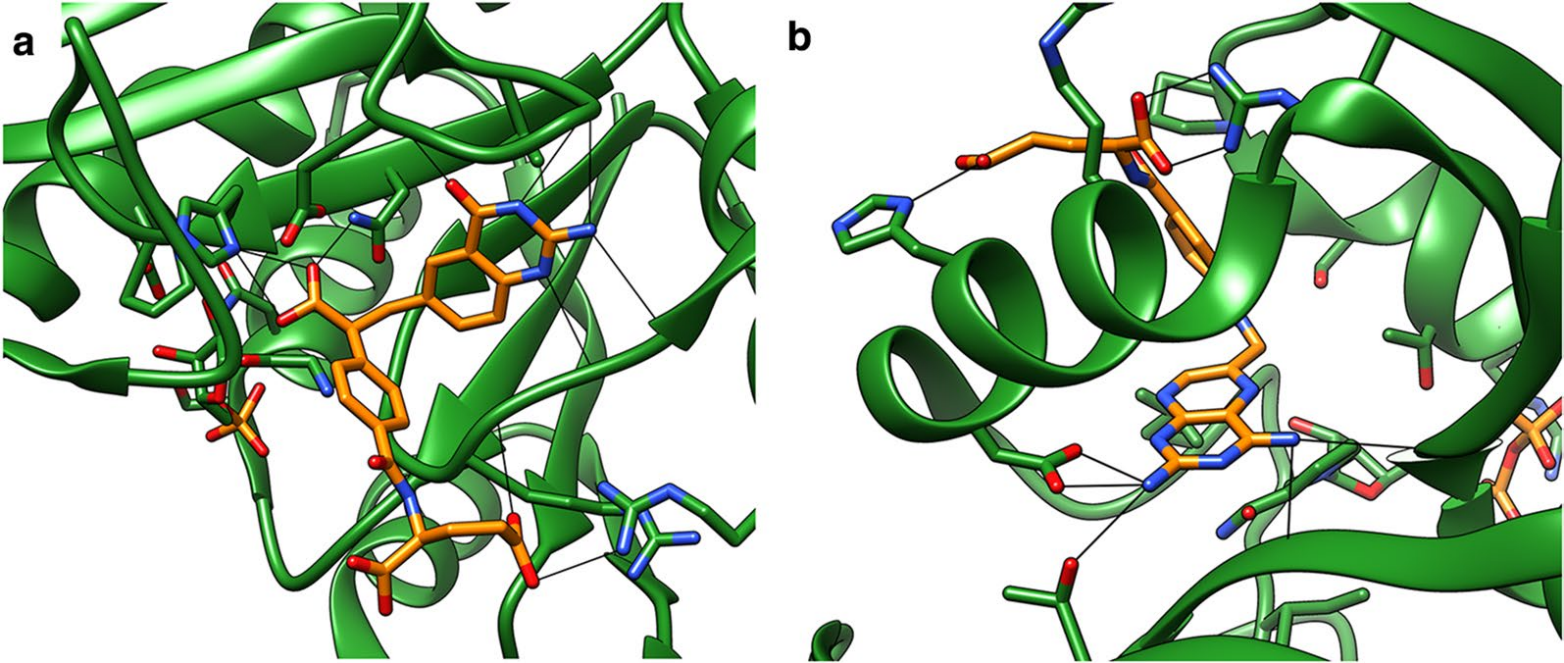
Hydrogen bonds and salt bridges in folate enzyme complexes. Representative complexes for **a** GART (1c2t@pdb) and **b** DHFR (3dfr@pdb) show that ligands predominantly interact with the binding site via hydrogen bonds and salt bridges

**Fig. 3:**
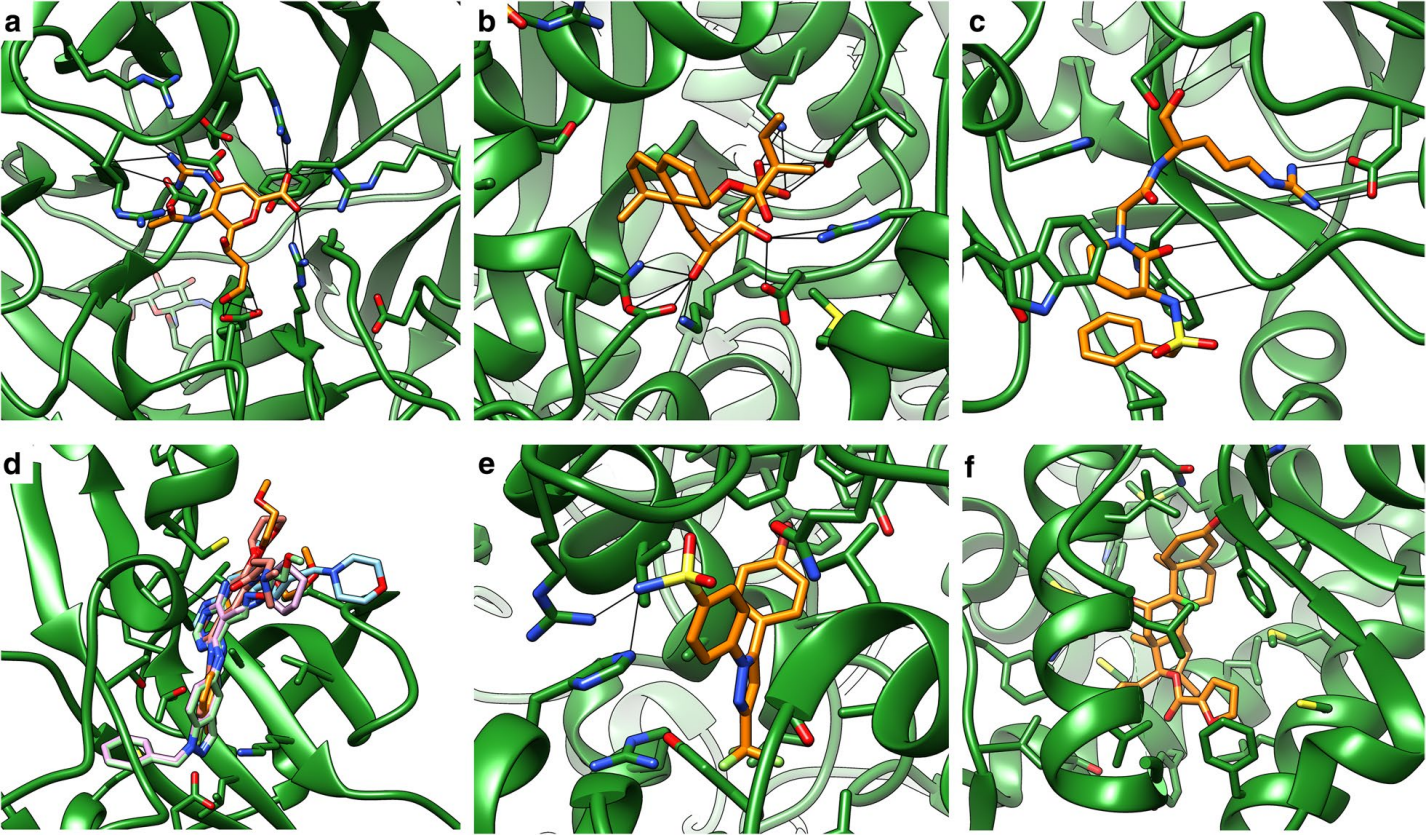
Interactions in binding sites of proteins with good interaction fingerprint performance. Representative complexes for **a** NA (1a4 g@pdb), **b** HMGR (1hw8@pdb), **c** thrombin (1ba8@pdb), **d** EGFR [overlay of 1m17 with ligands from 1m17 (orange), 1ax9 (blue), 2rgp (purple), 3bel (green) and 4g5j (red)], **e** COX-2 (1cx2@pdb) and **f** PR (1sr7@pdb)

A closer look into the several protein classes also revealed some general trends for the performance of PADIF scoring and interaction fingerprint scoring in general: For the folate enzymes, all methods yield very good results. This is likely due to the fact that the interactions in the respective binding sites involve a lot of specific hydrogen bonds and salt bridges with the ligands (Fig. 2). These are often easier to capture by computational methods than less directed nonpolar interactions. The same holds true for NA (Fig. 3a), HMGR (Fig. 3b), GPB, PNP and SAHH.

For DHFR, GART, HMGR, NA and PNP, many of the ligands in the used reference complexes show a high affinity in the nanomolar range, implying that the respective interactions are important and should also be observed in complexes with other actives. IFP and PADIF also perform well for the serine proteases. For this group, many ligands are designed to mimic the binding mode of the peptide substrates [29] (Fig. 3c). Thus, most of them are quite large and undergo certain important hydrogen bonds (for instance with a catalytic residue), resulting in a fingerprint with many non-zero elements that is suitable for a meaningful differentiation.

For kinases and for the other enzymes, scoring success of the interaction fingerprint methods strongly depends on the dataset. An ideal protein for fingerprint scoring is EGFR. The inhibitors in its reference complexes are relatively large, highly potent, exhibit many interactions with the binding site and additionally share quite similar binding modes with a good overlap of functional groups (Fig. 3d). A similar case with good overall performance is COX-2 (Fig. 3e). Here, the ligands in the reference complexes are also potent, showing both nonpolar interactions and hydrogen bonds.

A problem for some of the other datasets on which both ChemPLP and the fingerprint methods yield relatively bad enrichment might be protein flexibility. For ALR2 and FGFR1, conformational changes in the binding site upon binding of different ligands were observed [30, 31]. Standard docking cannot account for such induced fit phenomena, so that likely many of the resulting poses do not correspond to the true binding mode of the respective ligands. This makes rescoring via interaction fingerprints rather futile.

The nuclear hormone receptors are a particularly interesting protein group for fingerprint scoring since many ligands mimic natural substrates. Because of this, molecules are often similar and undergo similar interactions, which should be beneficial for interaction fingerprint approaches. Furthermore, binding sites of nuclear hormone receptors are rather hydrophobic [32], which might be difficult for conventional docking since nonpolar interactions are often only approximated by steric complementarity of atoms [33]. Indeed, the fingerprint approaches perform quite well for ER, mineralcorticoid receptor (MR) and RXR. For GR, a significant overall improvement is achieved by IFP, and a good early enrichment despite a low AUC can be also observed for the other fingerprint methods. In this case, the reduced overall performance might be due to the fact that all reference complexes contained ligands with a steroid-like scaffold, thus strongly biasing the scoring towards similar actives while neglecting others. For PR (Fig. 3f), a massive improvement can be seen compared to ChemPLP for the PADIF approaches. These findings indicate that for the nuclear hormone receptor datasets, ChemPLP was often not able to properly score the rather lipophilic ligands, but that interaction fingerprint scoring could compensate this insufficiency to some extent.

**Fig. 4:**
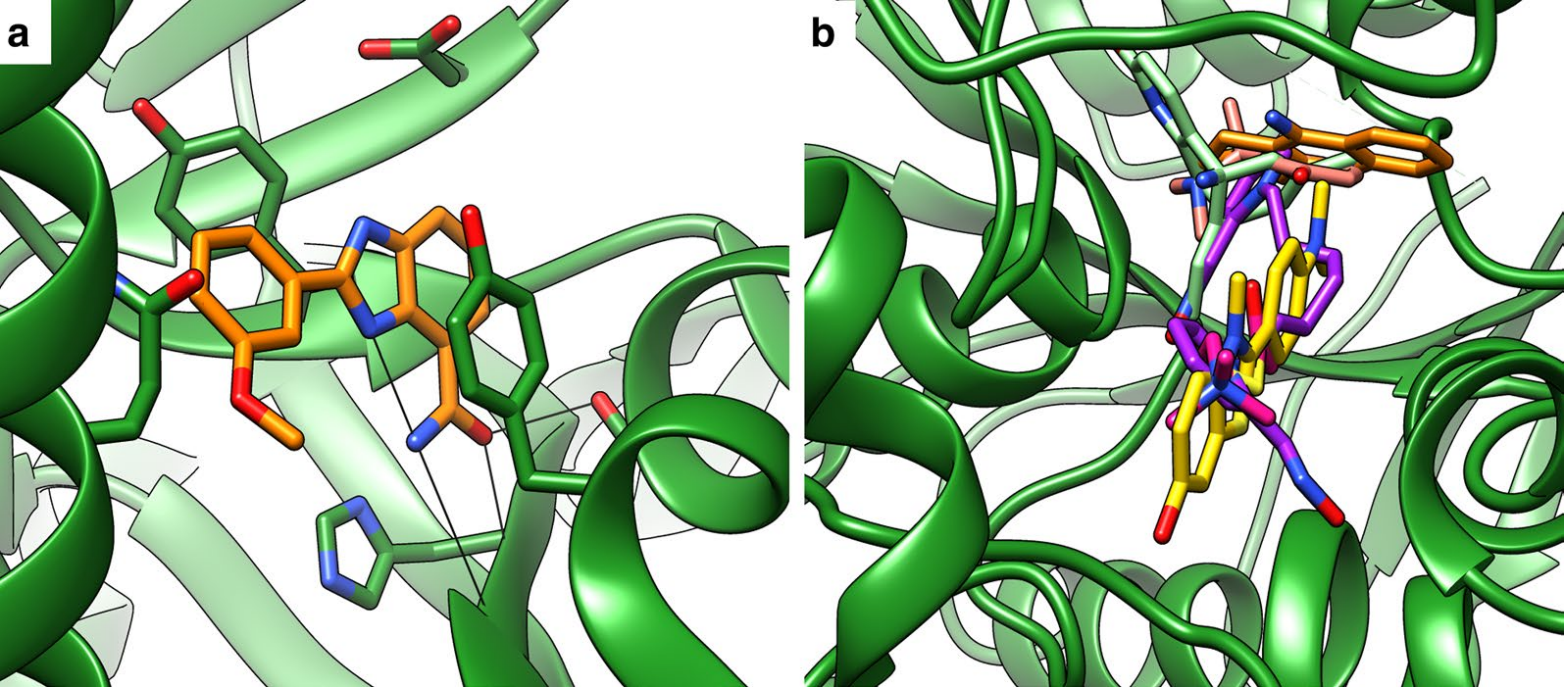
Proteins that are problematic for interaction fingerprint scoring: **a** PARP (1efy@pdb) has small ligands making only few interactions; **b** AChE [1acj@pdb, with overlay of ligands from 1acj (orange), 2j3q (yellow), 5bwc (violet), 1gpk (green) and 1gqr (red)] has structurally dissimilar ligands that bind in different parts of the binding site

A case which shows a logical limitation of interaction fingerprint scoring is the PARP dataset: Here, the fingerprint methods lead to a reasonable overall enrichment but show much worse early enrichment than ChemPLP. A reason might be that the available reference ligands are very small (average molecular mass approx. 194 g/mol) and thus undergo a limited number of interactions, including two hydrogen bonds (Fig. 4a). As a result, the reference fingerprints are small and all poses in which a molecule undergoes similar interactions get relatively high scores, so that a highly specific differentiation between actives and decoys directly at the beginning of the ranking is hardly possible.

Another example which is for rational reasons hard to tackle with interaction fingerprints is the dataset for AChE. AChE is a protein with very diverse ligands which are different in size and structure and exhibit diverse binding modes, sometimes even binding in different parts of the binding site (Fig. 4b). In addition, many of the active molecules are small with about one quarter having a molecular mass ≤ 250 g/mol. Nevertheless, combination of PADIF and ChemPLP can further improve the early enrichment for AChE, which demonstrates the robustness of the method.

**Fig. 5:**
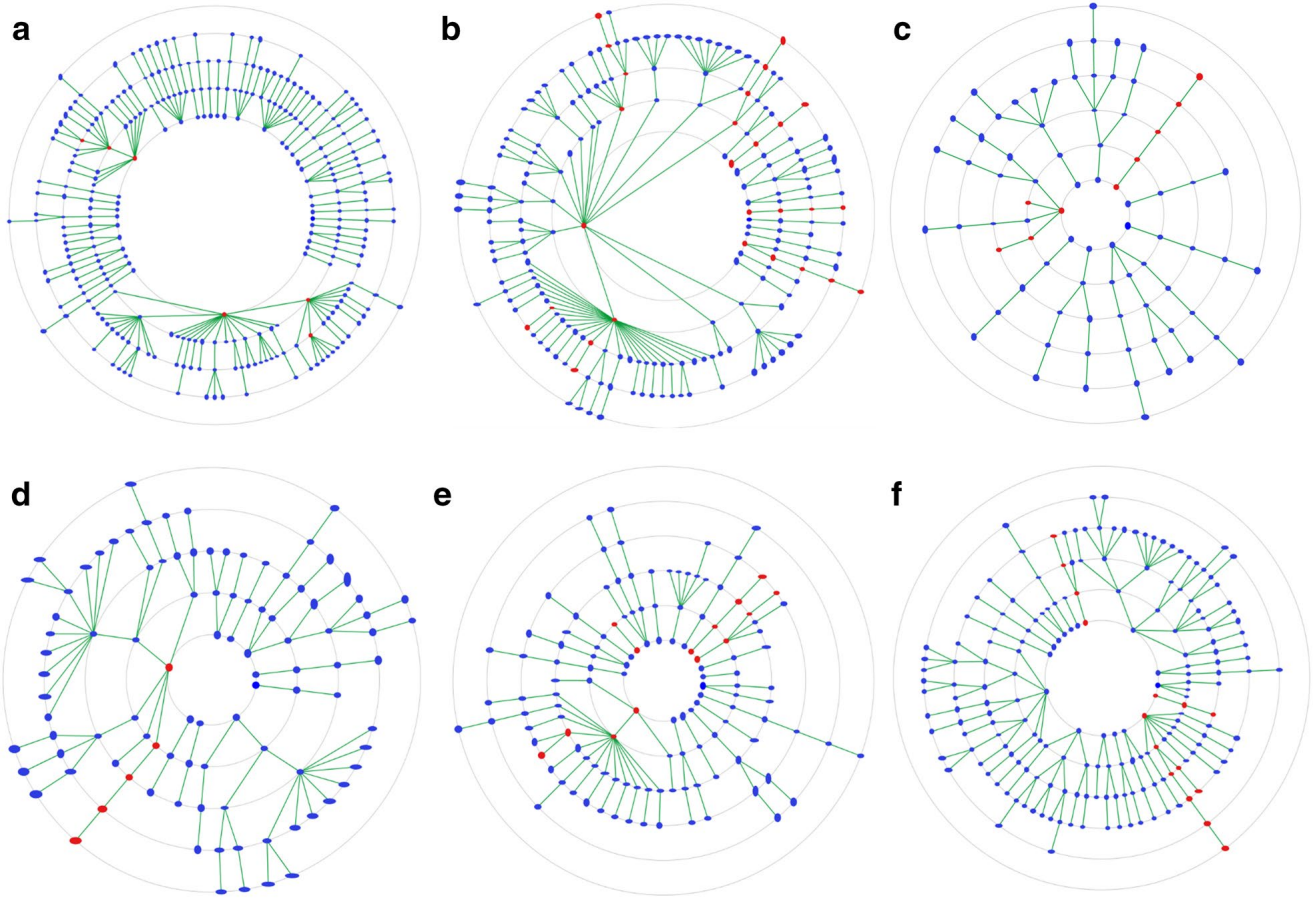
Scaffold tree. Hierarchical tree of the scaffolds in the DUD datasets and reference ligands for **a** COX-2, **b** EGFR, **c** ER (antagonist), **d** SRC, **e** thrombin and **f** P38. Scaffolds only present in ligands from the DUD dataset are shown in blue and scaffolds also present in the reference ligands in red. Figure created with Scaffold Hunter [34]

As could be expected from an interaction based approach, scoring success does not seem to rely on similarity of the underlying scaffolds: For the datasets of COX-2, EGFR, ER (antagonist), SRC, thrombin and P38, on which PADIF performed reasonably to excellent, the diversity of scaffolds in the active ligands and the used references was analysed with Scaffold Hunter (Fig. 5) [34]. The illustration shows a hierarchical tree of all occurring scaffolds, reaching from a one ring core scaffold in the inner sphere to up to six ring scaffolds in the outer sphere. Scaffolds only present in the DUD datasets are marked in blue and those also present in the references in red. Obviously, the fact that the actives contain a variety of scaffolds not present in the references does not affect scoring, which underlines the promising scaffold hopping potential of interaction fingerprint methods.

Furthermore, the potential influence of dataset diversity on PADIF scoring success was evaluated: For each protein dataset, the pairwise ECFP4 [35] Tanimoto similarities between all ligands were calculated [36] and binned (Additional file 1: S4). The distribution reveals that most pairwise similarities are rather low with over 80% being < 0.5 and over 60% being < 0.3, suggesting that most datasets are rather diverse. To find out if PADIF scoring performance correlates with the diversity of a dataset, the PADIF AUC values of the different protein datasets were plotted versus the respective percentage of pairwise similarities ≥ 0.5 and ≥ 0.7 (Additional file 1: S4). The plots show no significant correlation, and also for the other fingerprint methods no obvious correlation can be observed. This implies that fingerprint scoring is rather independent of the structural diversity of the dataset.

All in all, the findings of our study allow for some rules of thumb about the applicability of interaction fingerprint methods. In general, fingerprint scoring yielded especially good results when the ligands in known complex structures bind in the same regions of the binding site, exhibit similar interactions and show a high affinity. When it comes to different types of interactions, the results suggest that the involvement of many hydrogen bonds and salt bridges is beneficial for interaction fingerprint scoring. Although conventional scoring usually performs well in these cases, additional usage of fingerprint methods might still be useful for further enhancing the early enrichment. This can be seen for GART in case of the PADIF approaches. Huge improvement compared to conventional scoring can be achieved for challenging binding sites like for nuclear hormone receptors such as PR. However, special care should be taken when selecting the references: In order not to bias the results toward a very special compound class, it is sensible to select a set of references with rather diverse ligands. This introduces a certain variability into the fingerprints that is likely beneficial for finding ligands with new scaffolds.

## Conclusion

By evaluating the performance of three different types of interaction fingerprints for docking based virtual screening, it was demonstrated that interaction fingerprint scoring is in several cases able to further improve the results of the GOLD scoring function ChemPLP. Our study implies that classical, binding site specific interaction fingerprints are best suited for standard virtual screening. The PADIF approach utilises the protein per atom score contributions of the GOLD scoring functions, enables a multiple reference scoring with weighting and showed superior performance. This indicates that a quantitative fingerprint and the incorporation of the knowledge stored in more than one reference structure are beneficial for scoring. With respect to the applicability of fingerprint methods, our findings imply that the additional use of such methods is most promising for proteins for which many complexes with ideally highly potent inhibitors are available that exhibit specific interactions. Thus, results might even be improved for binding sites that are challenging for conventional scoring. On the other hand, care has to be taken for large binding sites in which different parts can be occupied by ligands.

For further improving the success of interaction fingerprint scoring in the future, it might be useful to expand the PADIF approach to the scoring functions of other docking programs and also to transfer the underlying concept of frequency weighted, multiple reference scoring to other interaction fingerprints. Furthermore, it could be beneficial to completely shift away from conventional similarity metrics and rather classify the fingerprints of docking poses by means of trained neural networks. For such applications, fingerprints like PADIFs might be especially suitable because they are not binary but contain float values representing the strength of the interactions (as estimated by the conventional scoring). In our scoring scheme, we did not fully exploit this stored knowledge and only differentiated between favourable and unfavourable interactions, but for machine learning methods this additional information might prove valuable.

## Methods

### PADIF based scoring

#### PADIF generation

PADIFs were derived from the protein per atom score contributions of the GOLD default scoring function ChemPLP either from rescoring files (experimental complexes) or GOLD solutions files (docking poses). These are exported by GOLD for the binding site atoms defined in the “cavity.atoms” file. The PADIFs have the dimension N × 8, where N is the number of binding site atoms and 8 is the number of interaction terms [ChemScore_PLP.Hbond, ChemScore_PLP.CHO, ChemScore_PLP.Metal, PLP.S(*hbond*), PLP.S(*metal*), PLP.S(*buried*), PLP.S(*nonpolar*) and PLP.S(*repulsive*)]. Depending on the contributions, the respective float values have different prefixes: for ChemScore_PLP.Hbond, ChemScore_PLP.CHO and ChemScore_PLP.Metal, positive values represent favourable interactions, for the other contributions negative values represent favourable interactions. For easier processing, prefixes of ChemScore_PLP.Hbond, ChemScore_PLP.CHO and ChemScore_PLP.Metal are reversed in the PADIF generation process, so that negative values always represent favourable interactions.

#### Calculation of a single reference PADIF

After the individual reference PADIFs are extracted from the GOLD rescoring files, they are combined into a single, median reference PADIF. Therefore, for each PADIF element the median of all respective values in the reference PADIFs is calculated for negative values so that only favourable interactions are considered. In case all reference values for a certain element are 0 or positive, the respective element value is set to 0. In addition, a weighting matrix is generated which assigns weighting factors to the elements depending on how often the respective interaction occurs in the reference PADIFs (for instance, if it occurs in four of ten complexes, the weighting factor is 0.4).

#### Scoring

The PADIF based scoring obeys the following scheme:

1. Determine the *R* elements (m, n) whose value is < 0 in the reference PADIF (favourable reference interactions).
2. Determine the *P* elements (m, n) whose value is < 0 in the pose PADIF (favourable interactions in the pose fingerprint).
3. Calculate the maximum possible Overlap *O*_max_ between reference and pose PADIF:
  $$O_{\text{max} } = P/R,\;{\text{but}}\;{\text{at}}\;{\text{maximum}}\; 1.$$
4. For the *R* elements (m, n) check the respective values in the pose PADIF and determine the individual elements score *S*(m,n) as following:
  *S*(m, n) = *w*(m, n) if pose PADIF(m, n) < 0.
  *S*(m, n) = 0 if pose PADIF(m, n) = 0.
  *S*(m, n) = − *w*(m,n) if pose PADIF (m, n) > 0.
5. Calculate the actual Overlap *O*_real_:
  $$O_{\text{real}} = \left( {P \cap R} \right)/R.$$
6. Calculate the relative overlap *O*_rel_:
  $$O_{\text{rel}} = O_{\text{real}} /O_{\text{max} } .$$
7. Calculate the total score *S*_tot_ by summing up the individual scores of all elements (for many unfavourable interactions, it might be a negative value) and decrease the total score depending on the deviation to a perfect overlap of 1.0:
  $$S_{\text{tot}} = \varSigma S\left( {m,n} \right){-}\left( {1.0 - O_{\text{rel}} } \right)\cdot\left| {\varSigma S\left( {m,n} \right)} \right|$$.

For the combination with ChemPLP, the ranking first contains only the best three percent of poses by ChemPLP followed by the PADIF based ranking of the rest. The purpose of this was to combine the strength of both methods to yield a very good early enrichment.

### Implementation

The PADIF based scoring was implemented in Java.

### IFP and TIFP scoring

#### IFP

IFP is a SIFt-like fingerprint that incorporates more interaction types than the original SIFt (for instance aromatic face to face or edge to face, weak H bonds, π cation or metal complexation) [12]. For IFP similarity calculations, default settings were kept. As reference, the native ligand of the protein structure used for docking was chosen.

#### TIFP

The fingerprint TIFP was tested as a representative for a binding site independent interaction fingerprint. In this approach, interactions are detected based on the pharmacophoric types of interacting protein and ligand atoms, resulting in so called interaction pseudoatoms. Possible triplet combinations within different distance ranges are counted and the full integer vector is pruned [17]. For TIFP similarity calculations, the fingerprint generation and comparison functionalities as implemented by the developers were automatized using an in-house python script. The pruned 210 integer version of the fingerprint was used; otherwise default settings were kept. Multiple reference complexes were used (all that were also applied for PADIF and IFP), resulting in one similarity based ranking for each reference complex.

### Targets, ligand data sets and reference complexes

#### Pose prediction

The Astex diverse dataset [24], comprising high resolution protein ligand complexes with drug like molecules and pharmaceutically relevant protein targets, was used to validate the PADIF approach for pose prediction. For a suited subset of 61 of these 85 complexes, appropriate reference complexes were selected from the Protein Data Bank (PDB) [37]. The other complexes of the Astex diverse dataset were excluded as either no other complex structures of the respective protein (at 100% sequence similarity) were available or the available structures contained ligands whose structural features and/or binding mode differed massively from that of the query ligand. Furthermore, some complexes were excluded as the respective ligands were too similar to the query ligand and hence would bias the results. In order to analyse a potential impact of the structural similarity between the molecules, the average ECFP4 and MDL Tanimoto similarity of the reference ligands and the query ligand were calculated using Pipeline Pilot [36]. The RMSD values between the docking poses and the native binding mode were calculated with fconv [38]. The PDB IDs of the reference complexes as well as the corresponding similarity values can be found in the Additional file 1: S2).

#### Virtual screening

39 proteins of the DUD dataset were used for virtual screening experiments; PDGFrb was excluded as the dataset only contained a homology model and no reference complex structures were available. For each protein, appropriate reference complexes were selected from the PDB. The IDs of the reference complexes as well as corresponding structural similarity values of the docked ligands to the reference ligands can be found in the Additional file 1: S3.

### Preparation of molecules and protein structures

Preparation of molecules was carried out using the program MOE [39]. They were first protonated using the “wash” function with the option “scale to reasonable bond length” enabled. After that they were minimised using MOE standard settings with the option “add hydrogens” disabled and the option “preserve existing chirality” enabled. Ligands from the Astex diverse dataset were used as provided.

Proteins were also prepared in MOE. Redundant chains, water molecules and ions were deleted (except for certain conserved water molecules in ADA and PDE5). Cofactors were kept except when reference ligands replaced at least parts of them. Protonation was carried out using the function “protonate 3D”. After that, possible corrections were made with the option “correct” after manual inspection.

### Docking

Docking experiments were carried out using GOLD with the default scoring function ChemPLP [19, 21]. Deviant from standard settings, the options “allow early termination” (Fitness and Search Options) and “Detect cavity—restrict atom selection to solvent-accessible surface” (Define Binding Site) were disabled. The options “flip pyramidal N”, “flip amide bonds” and “flip ring corners” (Ligand Flexibility) were enabled. If water molecules were present, the water option was set to “toggle”. For the docking experiments for binding mode predictions, the option “generate diverse solutions (1.5 Å)” (Fitness and Search Options) was used to ensure that a variety of diverse poses was generated. Search efficiency was set to 100% and the number of genetic algorithms was 100. The binding site was defined on the basis of used reference ligands with a radius of 10 Å. Besides, the options “write cavity atoms to file” and “save per atom scores” were enabled in all cases as a list of the cavity atoms and the per atom scores are needed for the PADIF scoring.

### Rescoring of reference complexes

For the datasets of the DUD, all used reference complexes were rescored using GOLD after the necessary preparation as described above. Atom numbering needs to be identical for PADIF scoring. However, even for PDB structures of the same protein, atom numbering usually differs. Thus, the respective rescore files were renumbered with a python script to match the atom numbering in the structure used for docking. Due to the high number of needed reference complexes, this procedure was simplified for the Astex complexes: Instead of preparing and rescoring every individual complex, all reference complexes as well as the protein used for docking were aligned and superposed using MOE. The resulting superposition was inspected manually in order to identify possible differences in sidechain conformations that might lead to atom clashes. The reference ligands and the protein chain used for docking were then rescored in GOLD.

### Assessment of active/decoy differentiation

For assessing the virtual screening performance of the tested methods, the AUC as well as the EF_1%_ and EF_3%_ were calculated using the ROC Curve and Virtual Screening Metrics nodes in Knime [40].

### Graphics

Graphics of protein structures and ligands were generated with Chimera [41].

## Additional file

**Additional file 1.** The file provides further information related to this article as supplemental material.

## Abbreviations

PADIF: protein per atom score contributions derived interaction fingerprint
SIFt: structural interaction fingerprint
SPLIF: structural protein ligand interaction fingerprint
APIF: atom pairs based interaction fingerprint
ECFP: extended connectivity fingerprints
DUD: directory of useful decoys
RMSD: root mean square deviation
ROC: receiver operating characteristic
AUC: area under the ROC curve
MDL: molecular design limited
PDB: protein data bank

## Proteins

ACE: angiotensin converting enzyme
AChE: acetylcholine esterase
ADA: adenosine deaminase
ALR2: aldose reductase 2
AmpC: ampicillin β-lactamase
COX-1: prostaglandin H(2) synthase-1 (containing COX-1)
COX-2: cyclooxygenase 2
EGFR: epidermal growth factor receptor
ER: estrogen receptor
FGFR1: fibroblast growth factor receptor 1
FXa: factor Xa
GR: glucocorticoid receptor
HMGR: HMG-CoA reductase
MR: mineralcorticoid receptor
NA: neuraminidase
PARP: poly (ADP-ribose) polymerase
PPAR: peroxisome proliferator activated receptor
PR: progesterone receptor
RXR: retinoid X receptor
SRC: cellular sarcoma tyrosine kinase
TK: thymidine kinase
VEGFR2: vascular endothelial growth factor receptor
