# Supplementary material for: A novel interaction fingerprint derived from per atom score contributions: exhaustive evaluation of interaction fingerprint performance in docking based virtual screening
Source: J Cheminform. 2018 Mar 16;10:15. doi: 10.1186/s13321-018-0264-0 (PMC5856854; doi:10.1186/s13321-018-0264-0)
Supplement: Supplementary file 1 — Additional file 1. The file provides further information related to this article as supplemental material. [file 13321_2018_264_MOESM1_ESM.pdf]

A Novel Interaction Fingerprint Derived from Per Atom Score Contributions: Exhaustive Evaluation of Interaction Fingerprint Performance in Docking Based Virtual Screening

Julia B. Jasper[1] (julia.jasper@tu-dortmund.de), Lina Humbeck[1] (lina.humbeck@tu-dortmund.de), Tobias Brinkjost[1,2] (tobias.brinkjost@tu-dortmund.de), Oliver Koch[1]* (oliver.koch@tu-dortmund.de)

[1]Faculty of Chemistry and Chemical Biology, TU Dortmund University, Otto-Hahn-Str. 6, 44227 Dortmund, Germany

[2]Department of Computer Science, TU Dortmund University, Otto-Hahn-Str. 14, 44227 Dortmund

Supplemental Material

S1 – A Specific Scoring Scheme for Binding Mode Prediction

Overview. In addition to the scoring scheme described in our study, we also developed another one which was especially designed for a very exact binding mode prediction rather than the relative ranking of different molecules. It does not only differentiate between favourable and unfavourable interactions but compares the quantitative strength of each pose fingerprint interaction to the respective median value of the combined references. This scoring procedure indeed showed a very good pose recovery power, so that 61 % and 80 % of the top ranked poses for the Astex diverse dataset complexes showed an RMSD below 1.0 Å and 2.0 Å, respectively. An analysis of the correlation between the RMSD of the top ranked pose and the average Tanimoto ECFP4 and MDL similarity between the reference ligands and the query molecule (Fig. S1) confirmed that scoring success does not correlate with structural similarity, as would be expected for an interaction fingerprint.

Scoring Scheme. The pose recovery scheme does not only differentiate between favourable and unfavourable interactions but also exploits the quantitative values in the PADIFs and uses slightly different weighting factors to weight very important interactions even stronger (10, 4, 2, 1 for a relative occurrence in > 75, 50, 30 and 0 percent of complexes, respectively) (Tab. S1). Unfavourable

interactions (case 1) lead to a negative PADIF score, with the absolute value depending on the quantitative value of the contribution and the weighting factor of the element. Missing interactions (case 4) are also penalized depending on the weighting factors. Absence of interactions which are also absent in the reference (case 2) are slightly rewarded, as well as the presence of additional interactions not present in the references (case 3). Interactions similar to the reference (case 5) are rewarded taking into account both the strength of the interaction in relation to the reference and the respective weighting factor.

Table S1. Rules for calculating the PADIF scoring in a scheme especially for binding mode prediction.

Case	Condition	Calculation of element score *S*(m,n)
**1**	Pose PADIF(m,n) > 0	S(m,n) = (-1)·pose PADIF(m,n)·(1 + w(m,n))
**2**	Reference PADIF(m,n) = 0,<br>Pose PADIF(m,n) = 0	S(m,n) = 0.2
**3**	Reference PADIF(m,n) = 0,<br>Pose PADIF(m,n) < 0	S(m,n) = pose PADIF(m,n), but maximum 0.3
**4**	Reference PADIF(m,n) < 0,<br>Pose PADIF(m,n) = 0	S(m,n) = -1.2·w(m,n)
**5**	Reference PADIF(m,n) < 0,<br>Pose PADIF(m,n) < 0	$ratio = \frac{\text{pose PADIF(m,n)}}{\text{ref. PADIF(m,n)}}$, but maximum 1.2<br>$S$(m,n) = $ratio \cdot w$(m,n)

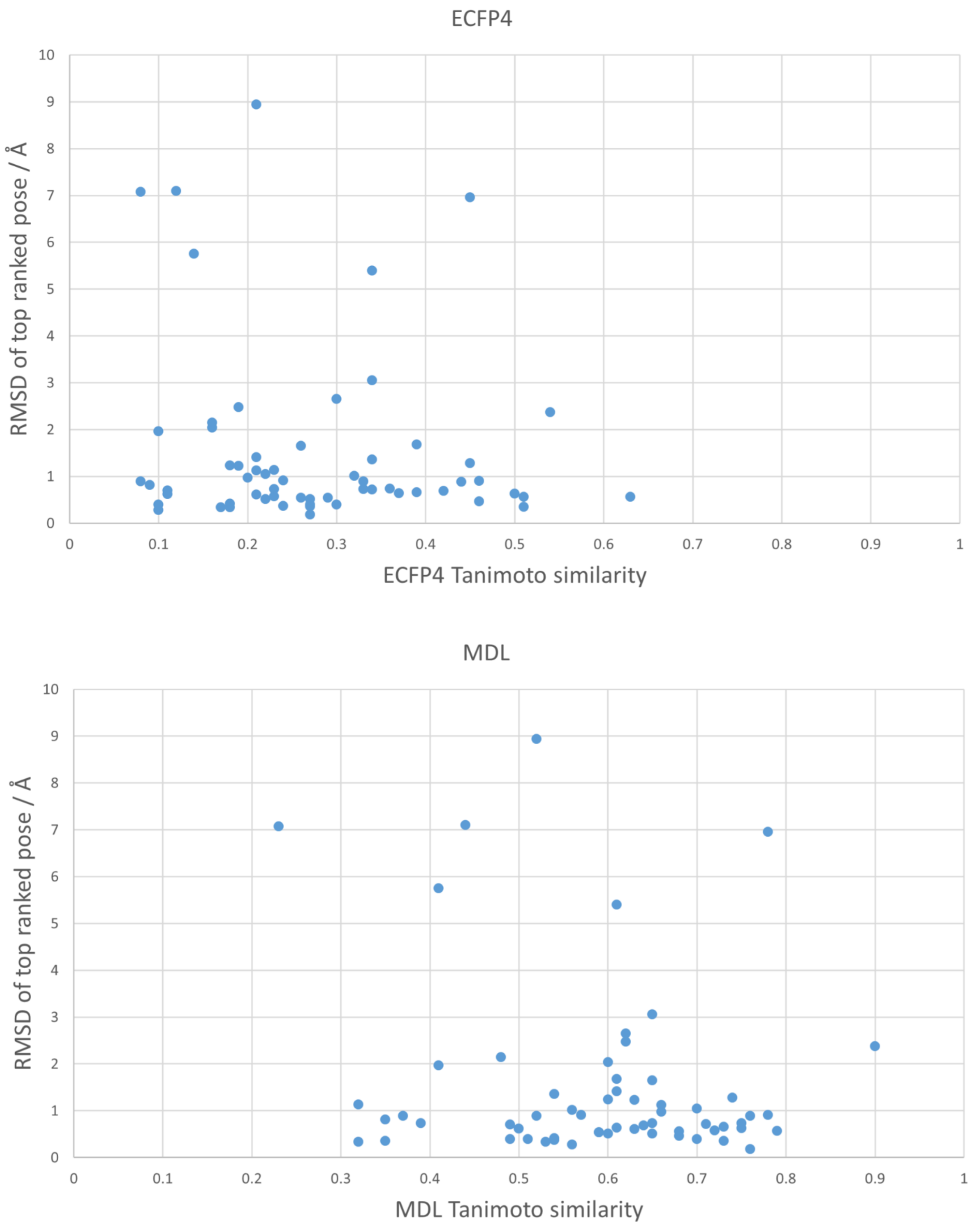

Figure S1: Correlation between RMSD and compound similarity for the Astex dataset. Plot of the RMSD value of the top ranked pose versus the respective a) average ECFP Tanimoto similarity and b) average MDL Tanimoto similarity between the query ligand and the reference ligands for the adapted pose recovery scoring.

S2 - Reference complexes for pose recovery

Table S2: Complexes from the Astex diverse dataset with the respective reference structures and the mean ECFP4 and MDL Tanimoto similarity of the reference ligands with the query compounds.

PDB Code	Reference structures	Ø T_c-ECFP4	Ø T_c-MDL	Protein
**1gpk_HUP**	1gpn_HUB, 3zv7_NHG	0.26 ± 0.16	0.59 ± 0.18	acetylcholine esterase (AChE)
**1hnn_SKF**	1n7i_LY1, 1n7j_IDI, 2an3_CTL, 2an5_TTL, 3kpw_1SQ, 3kpy_ES2	0.23 ± 0.14	0.32 ± 0.06	phenylethanolamine N-methyltransferase (PNMT)
**1hww_SWA**	3dx1_YHO, 3ddf_GB6, 2ow6_NK1, 1tqw_BLT, 1qwn_GUL	0.10 ± 0.06	0.56 ± 0.11	golgi α mannosidase II
**1hwi_115**	2q6b_HR2, 2q6c_HR1, 2r4f_RIE, 3ccz_5HI, 1hw8_114	0.34 ± 0.08	0.61 ± 0.10	HMG-CoA reductase (HMGR)
**1ia1_TQ3**	1m79_MQ1, 1m78_CLZ, 1m7a_MQU, 3qls_55V, 1ia3_TQ5, 1aoe_GW3	0.27± 0.18	0.60 ± 0.11	dihydrofolate reductase (DHFR)
**1ig3_VIB**	2f17_PYI	0.42	0.64	thiamine pyrophosphokinase (TPP)
**1jd0_AZM**	4ht2_V50, 4qjw_WWO, 4kp5_E1F, 4kp8_E1G	0.16 ± 0.02	0.60 ± 0.06	carbonic anhydrase XII

**1jje_BYS**	1jjt_BDS, 4c1f_X8Z, 1dd6_MCI, 3wxc_C93	0.34 ± 0.30	0.54 ± 0.27	IMP-1 metallo β-lactamase
**1jla_TNK**	3drr_R8E, 1jkh_EFZ	0.08 ± 0.01	0.37 ± 0.03	HIV-1 reverse transcriptase (HIVRT)
**1k3u_IAD**	1kfc_IPL, 3pr2_F9F	0.23 ± 0.13	0.39 ± 0.07	tryptophane synthase
**1ke5_LS1**	4fku_60K, 4gcj_X64	0.23 ± 0.06	0.72 ± 0.06	cyclin dependant kinase II (CDKII)
**1kzk_JE2**	3el1_DR7, 3i7e_DJR, 3aid_ARQ	0.18 ± 0.05	0.54 ± 0.02	HIV JE-2147 protease (HIVPR)
**1l2s_STC**	4kzb_NZ2, 4kza_NZ9, 4e3l_0NB, 4e3k_0NA	0.26 ± 0.17	0.65 ± 0.10	AmpC β-lactamase
**1l7f_BCZ**	4dgr_3LV, 1f8e_49A, 1f8c_4AM, 1nnc_ZMR, 2qwk_G39, 1bji_DPC	0.18 ± 0.04	0.53 ± 0.07	neuraminidase (NA)
**1lpz_CMB**	4y6d_48U, 2y81_931, 1lpg_IMA, 1lpk_CBB, 1f0s_PR2, 2boh_IIA, 1lqd_CMI	0.29 ± 0.26	0.59 ± 0.15	factor Xa (FXa)
**1mmv_3AR**	1qwc_14W, 4fvw_1KJ, 1k2s_ARV, 5agl_CCW	0.39 ± 0.21	0.61 ± 0.17	neuronal NOS heme domain
**1mzc_BNE**	1s63_778, 1ld7_U66, 1ld8_U49	0.19 ± 0.02	0.62 ± 0.01	farnesyl transferase
**1n1m_A3M**	3vjm_W61, 2g63_AAF, 2g5t_ACF, 2ogz_U1N, 2i03_AXD	0.11 ± 0.01	0.50 ± 0.07	dipeptidyl peptidase IV/CD26

**1n2v_BDI**	4q4s_S98, 1r5y_DQU 1p0b_PQO	0.11 ± 0.04	0.49 ± 0.03	tRNA guanine transglycosylase (TGT)
**1n46_PFA**	3gws_T3, 3jzc_4HY, 1nax_IH5, 3imy_B72, 1q4x_G24	0.27 ± 0.07	0.35 ± 0.02	thyroid receptor β (TR β)
**1nav_IH5**	4lnw_T3, 3jzb_4HY, 3ilz_B72	0.44 ± 0.06	0.76 ± 0.09	thyroid receptor α (TR α)
**1of1_SCT**	1e2k_TMC, 4oql_F2U, 3f0t_NCV, 2ki5_AC2, 4oqm_FDU	0.27 ± 0.10	0.76 ± 0.16	thymidine kinase (TK)
**1oq5_CEL**	2nno_M28, 2nnv_M29, 5byi_4WA, 3s8x_E59	0.24 ± 0.01	0.57 ± 0.07	carbonic anhydrase II
**1owe_675**	1owh_239, 1sqo_UI2, 1sqa_UI1	0.51 ± 0.14	0.73 ± 0.10	urokinase
**1oyt_FSN**	1y3w_UIP, 2cf8_ESH, 2zda_32U, 2zdl_45U	0.27 ± 0.06	0.70 ± 0.07	thrombin
**1p2y_NCT**	2h7q_IMD, 1phd_PIW	0.09 ± 0.07	0.35 ± 0.11	cytochrome P450 (CYP450)
**1p62_GEO**	1p5z_AR3, 3ipx_B86, 2a7q_CFB, 2qrn_DCM, 2pd4_DCN	0.34 ± 0.18	0.71 ± 0.25	deoxycytidine kinase (dCK)
**1pmn_984**	3oy1_589, 4y46_4F2, 4z9l_880, 3ttj_JBI, 4kkh_1RQ	0.18 ± 0.04	0.60 ± 0.06	c-Jun terminal kinase 3 (JNK3)
**1q1g_MTI**	3phc_IM5, 1nw4_IMH	0.22 ± 0.02	0.70 ± 0.03	purine nucleoside

				phosphorylase (PNP)
**1q41_IXM**	4pte_2WF, 3say_OFT, 1q3d_STU, 1q3w_ATU, 1q4l_679	0.24 ± 0.19	0.54 ± 0.09	glycogen synthase kinase 3β (GSK 3β)
**1r1h_BIR**	1dmt_RDF, 2qpj_I20, 1y8j_STS, 1r1j_OIR	0.33 ± 0.14	0.52 ± 0.17	neprilysin
**1r58_AO5**	1r5g_AO1, 1r5h_AO2	0.34 ± 0.05	0.65 ± 0.00	methionine aminopeptidase 2 (MetAP2)
**1r9o_FLP**	5a5i_XI1	0.08	0.23	cytochrome P450 2C9
**1s19_MC9**	3b0t_MCZ, 3az3_DS6, 1ie9_VDX, 1ie8_KH1	0.46 ± 0.24	0.78 ± 0.15	vitamin D nuclear receptor (VDR)
**1s3v_TQD**	1kmv_LII, 1kms_LIH, 3ghw_GHW, 4g95_OAG	0.10 ± 0.03	0.49 ± 0.06	dihydrofolate reductase (DHFR)
**1sg0_STL**	3ox2_79X	0.17	0.32	quinone reductase 2 (QR2)
**1sj0_E4D**	2iog_IOG, 1xpc_AIT, 1xp6_AIU, 1xp1_AIH	0.63 ± 0.20	0.79 ± 0.07	estrogen receptor α (ER)
**1sqn_NDR**	1sr7_MOF, 1a28_STR, 2ovh_AS0	0.22 ± 0.06	0.65 ± 0.10	progesterone receptor (PR)
**1t40_ID5**	1mar_ZST, 2ine_PAC, 2inz_OHP, 1iei_ZES, 1ef3_FID	0.16 ± 0.02	0.48 ± 0.17	aldose reductase (ALR2)
**1t46_STI**	4u0i_OLI	0.39	0.73	c-KIT tyrosine kinase
**1tz8_DES**	4qxv_LU2, 4l1t_1WZ,	0.14 ± 0.01	0.41 ± 0.04	transthyretin

	3m1o_CJZ			
**1t9b_1CS**	1t9d,_1MM 1t9c_1SM, 1t9a_1TB, 1n0h_CIE	0.54 ± 0.11	0.90 ± 0.02	acetohydroxyacid synthase
**1tow_CRZ**	3fr4_F8A, 3fr5_I4A, 3fr2_8CA, 1tou_B1V	0.21 ± 0.06	0.66 ± 0.13	adipocyte fatty acid binding protein (A-FABP)
**1tt1_KAI**	1s9t_QUS, 1sd3_SYM, 4H8I_11W	0.12 ± 0.02	0.44 ± 0.01	GluR6-kainate receptor
**1uml_FR4**	1ndy_FR3, 1ndw_FR2, 1ndz_FR5, 2z7g_EH9, 2e1w_FR6	0.45 ± 0.21	0.78 ± 0.10	adenosine deaminase (ADA)
**1unl_RRC**	3o0g_300, 1ung_ALH, 1unh_IXM	0.10 ± 0.02	0.41 ± 0.07	cyclin dependant kinase 5 (CDK5)
**1v48_HA1**	1b8n_IMG, 1b8o_IMH, 2ai2_P1D, 2ai3_P2G, 2qpl_BTY	0.21 ± 0.11	0.61 ± 0.09	purine nucleoside phosphorylase (PNP)
**1v4s_MRK**	3fr0_AJB, 3goi_LOI, 3vev_OH4, 3vf6_OH6, 4no7_2N8	0.20 ± 0.10	0.66 ± 0.16	glucokinase
**1vcj_IBA**	1b9s_FDI, 1b9t_RAI, 1b9v_RA2, 1ivb_ST1	0.51 ± 0.27	0.68 ± 0.20	neuraminidase (NA)
**1w1p_GIO**	1e6r_AMI, 1e6z_NGO, 1o6i_0HZ, 1w1t_CHQ, 1w1v_ALJ, 1w1y_TYP	0.21 ± 0.14	0.63 ± 0.16	chitinase B
**1w2g_THM**	1mrs_5HU, 4unq_H6D, 1gtv_TYD, 1e98_ATM	0.45 ± 0.21	0.74 ± 0.18	thymidylate kinase

**1xm6_5RM**	1xm4_PIL, 1xlx_CIO, 1xlz_FIL, 1xn0_ROL 1xmu_ROF	0.21 ± 0.04	0.52 ± 0.06	phosphodiesterase 4B (PDE4B)
**1xoq_ROF**	1y2k_7DE, 1xon_PIL, 4w1o_3GJ	0.37 ± 0.18	0.61 ± 0.16	phosphodiesterase 4D (PDE4D)
**1y6b_AAX**	1y6a_AAZ, 3vid_4TT	0.32 ± 0.24	0.56 ± 0.26	vascular endothelial growth factor receptor 2 (VEGFR2)
**1ygc_905**	2bz6_346, 1w7x_413, 4yt6_4JY, 4zxy_4T1	0.30 ± 0.12	0.51 ± 0.08	factor VIIa (FVIIa)
**1yqy_915**	5d1s_56Q, 4pku_30P, 4wf6_407	0.50 ± 0.09	0.75 ± 0.03	lethal factor
**1yvf_PH7**	1z4u_PH9	0.36	0.75	RNA dependant RNA polymerase
**1z95_198**	2ax8_FHM	0.46	0.68	androgen receptor
**2bm2_PM2**	2zeb_11M, 2zec_11N, 3v7t_0GX, 4a6l_P43	0.30 ± 0.12	0.62 ± 0.05	beta-II tryptase
**2br1_PFP**	2brh_DFW, 2ydi_YDI, 2ym3_YM3, 3pa3_C70, 4qyg_3DW	0.19 ± 0.16	0.63 ± 0.10	Chk1 kinase
**2bsm_BSM**	2yi7_BZ8, 4lwe_FJ2, 3eko_PYU	0.33 ± 0.15	0.65 ± 0.12	heat shock protein 90 (HSP90)

S3 - Reference Complexes for Virtual Screening

Table S3: PDB Codes of the complexes used for docking and the reference complexes of PADIF based scoring together with the respective average ECFP4 and MDL Tanimoto similarities between 1) all reference ligands and all active molecules (Ref/A), 2) all reference ligands and all decoy molecules (Ref/D), 3) between all active molecules (A/A) and 4) between all decoy and active molecules (A/D).

PDB Code	Reference structure	Ø T_c-ECFP4	Ø T_c-MDL	Protein
1o86	1uze_EAL, 1uzf_MCO, 2oc2_RX3, 4bzr_K26	Ref/A: 0.14 ± 0.17<br>Ref/D: 0.11 ± 0.17<br>A/A: 0.27 ± 0.15<br>A/D: 0.15 ± 0.05	Ref/A: 0.19 ± 0.06<br>Ref/D: 0.19 ± 0.04<br>A/A: 0.57 ± 0.14<br>A/D: 0.48 ± 0.09	angiotensin converting enzyme (ACE)
1acj	1acl_DME, 1ax9_EDR, 1e3q_EBW, 1gpk_HUP, 1gqr_SAF, 1acj_THA, 2c58_AT3, 2j3q_TFL, 3zv7_NHG, 5bwc_HBP, 1e66_HUX	Ref/A: 0.07 ± 0.04<br>Ref/D: 0.07 ± 0.04<br>A/A: 0.30 ± 0.18<br>A/D: 0.15 ± 0.06	Ref/A: 0.38 ± 0.09<br>Ref/D: 0.38 ± 0.08<br>A/A: 0.58 ± 0.18<br>A/D: 0.53 ± 0.11	acetylcholine esterase (AChE)
1ndw	1ndw_FR2, 1v7a_FRC,	Ref/A: 0.15 ± 0.11<br>Ref/D: 0.10 ± 0.03	Ref/A: 0.57 ± 0.08	adenosine deaminase

	1v79_FR7, 1wxz_FRL, 2z7g_EH9	A/A: 0.22 ± 0.16<br>A/D: 0.09 ± 0.06	Ref/D: 0.43 ± 0.10<br>A/A: 0.62 ± 0.18<br>A/D: 0.44 ± 0.13	(ADA)
1ah3	1eko_I84, 1ah3_TOL	Ref/A: 0.11 ± 0.03<br>Ref/D: 0.12 ± 0.04<br>A/A: 0.18 ± 0.18<br>A/D: 0.11 ± 0.04	Ref/A: 0.39 ± 0.08<br>Ref/D: 0.37 ± 0.06<br>A/A: 0.48 ± 0.18<br>A/D: 0.42 ± 0.09	aldose reductase (ALR2)
1xgj	1l2s_STC, 4jxs_18U, 4jxv_1MU	Ref/A: 0.32 ± 0.13<br>Ref/D: 0.13 ± 0.05<br>A/A: 0.37 ± 0.17<br>A/D: 0.17 ± 0.06	Ref/A: 0.73 ± 0.15<br>Ref/D: 0.46 ± 0.12<br>A/A: 0.71 ± 0.20<br>A/D: 0.46 ± 0.13	AmpC β-lactamase (AmpC)
2ao6	1e3g_R18, 1t7r_DHT, 2am9_TES, 2amb_17H, 2ax9_BHM, 2axa_FHM, 2ihq_LG7, 3v4a_PK1, 3v49_PK0	Ref/A: 0.15 ± 0.13<br>Ref/D: 0.09 ± 0.04<br>A/A: 0.20 ± 0.16<br>A/D: 0.10 ± 0.04	Ref/A: 0.45 ± 0.22<br>Ref/D: 0.37 ± 0.12<br>A/A: 0.50 ± 0.22<br>A/D: 0.36 ± 0.11	androgen receptor (AR)
1ckp	1h00_FAP, 2b55_D31, 2r3f_SC8,	Ref/A: 0.11 ± 0.07<br>Ref/D: 0.11 ± 0.04	Ref/A: 0.47 ± 0.09	cyclin dependant kinase II

	2r3q_5SC, 3rpr_25Z, 4bzd_D6I, 4ek4_1CK, 4fkt_48K, 4gcj_X64	A/A: 0.11 ± 0.09<br>A/D: 0.10 ± 0.04	Ref/D: 0.44 ± 0.09<br>A/A: 0.48 ± 0.11<br>A/D: 0.46 ± 0.09	(CDK2)
1h1d	1jr4_CL4, 1vid_DNC, 2cl5_BIE, 3hvj_705, 3s68_TCW	Ref/A: 0.19 ± 0.09<br>Ref/D: 0.12 ± 0.06<br>A/A: 0.29 ± 0.22<br>A/D: 0.12 ± 0.05	Ref/A: 0.42 ± 0.14<br>Ref/D: 0.44 ± 0.11<br>A/A: 0.51 ± 0.17<br>A/D: 0.33 ± 0.12	catechol O-methyl-transferase (COMT)
1q4g	1eqg_IBP, 1ht8_34C, 1pth_SAL, 1q4g_BFL, 2ayl_FLP, 2oyu_IMS, 3kk6_CEL, 3n8y_DIF, 4o1z_MXM	Ref/A: 0.15 ± 0.07<br>Ref/D: 0.13 ± 0.04<br>A/A: 0.18 ± 0.13<br>A/D: 0.16 ± 0.05	Ref/A: 0.34 ± 0.12<br>Ref/D: 0.32 ± 0.10<br>A/A: 0.39 ± 0.15<br>A/D: 0.39 ± 0.11	prostaglandin H(2) synthase-1 (containing COX-1)
1cx2	4cox_IMN, 6cox_S58	Ref/A: 0.19 ± 0.14<br>Ref/D: 0.11 ± 0.04<br>A/A: 0.21 ± 0.10<br>A/D: 0.11 ± 0.04	Ref/A: 0.51 ± 0.19<br>Ref/D: 0.47 ± 0.09<br>A/A: 0.56 ± 0.15<br>A/D: 0.42 ± 0.11	cyclooxygenase 2 (COX-2)
3dfr	1bzf_TMQ, 1dis_BDM, 2hm9_TRR,	Ref/A: 0.22 ± 0.09<br>Ref/D: 0.11 ± 0.03<br>A/A: 0.24 ± 0.10	Ref/A: 0.65 ± 0.07<br>Ref/D: 0.47 ±	dihydrofolate reductase (DHFR)

	3dfr_MTX	A/D: 0.11 ± 0.05	0.08<br>A/A: 0.68 ± 0.09<br>A/D: 0.44 ± 0.10	
1m17	1m17_AQ4,<br>2ity_IRE, 2j6m_AEE,<br>2rgp_HYZ,<br>3bel_POX, 3poz_03P,<br>3w2s_W2R,<br>4g5j_0WN,<br>4i23_1C9, 5cav_4ZQ	Ref/A: 0.18 ± 0.07<br>Ref/D: 0.11 ± 0.03<br>A/A: 0.29 ± 0.14<br>A/D: 0.11 ± 0.04	Ref/A: 0.50 ±<br>0.09<br>Ref/D: 0.44 ±<br>0.09<br>A/A: 0.56 ± 0.12<br>A/D: 0.41 ± 0.09	epidermal<br>growth factor<br>receptor (EGFR)
2p15	2qgt_EED,<br>2p15_EZT	Ref/A: 0.25 ± 0.15<br>Ref/D: 0.09 ± 0.03<br>A/A: 0.22 ± 0.16<br>A/D: 0.09 ± 0.04	Ref/A: 0.57 ±<br>0.18<br>Ref/D: 0.30 ±<br>0.08<br>A/A: 0.53 ± 0.20<br>A/D: 0.28 ± 0.10	estrogen<br>receptor α (ER,<br>agonist)
1sj0	1sj0_E4D,<br>2bj4_OHT, 3erd_DES	Ref/A: 0.20 ± 0.08<br>Ref/D: 0.11 ± 0.04<br>A/A: 0.28 ± 0.18<br>A/D: 0.12 ± 0.04	Ref/A: 0.50 ±<br>0.17<br>Ref/D: 0.37 ±<br>0.10<br>A/A: 0.67 ± 0.16<br>A/D: 0.46 ± 0.10	estrogen<br>receptor α (ER,<br>antagonist)
1agw	1agw_SU2,<br>1fgi_SU1,<br>3wj6_LWJ,	Ref/A: 0.12 ± 0.05<br>Ref/D: 0.13 ± 0.04<br>A/A: 0.26 ± 0.20	Ref/A: 0.53 ±<br>0.10<br>Ref/D: 0.49 ±	fibroblast<br>growth factor<br>receptor 1

	4uwb_JVT, 4uwc_4YO, 4wun_66T, 4zsa_4UT, 5am6_380	A/D: 0.11 ± 0.03	0.09 A/A: 0.63 ± 0.15 A/D: 0.49 ± 0.09	(FGFR1)
1lpg	1lpg_IMA, 1nfw_RRR, 1z6e_IK8, 2pr3_237, 2xc4_IVK, 3cen_FXA, 3q3k_D90	Ref/A: 0.13 ± 0.08 Ref/D: 0.11 ± 0.04 A/A: 0.33 ± 0.19 A/D: 0.12 ± 0.04	Ref/A: 0.51 ± 0.11 Ref/D: 0.50 ± 0.09 A/A: 0.63 ± 0.16 A/D: 0.47 ± 0.09	factor Xa (FXa)
1c2t	1c2t_NHS, 1c3e_NHR1cde_DZF, 1gar_U89, 1jkx_138,	Ref/A: 0.23 ± 0.09 Ref/D: 0.14 ± 0.07 A/A: 0.46 ± 0.11 A/D: 0.19 ± 0.12	Ref/A: 0.64 ± 0.10 Ref/D: 0.53 ± 0.11 A/A: 0.84 ± 0.08 A/D: 0.54 ± 0.13	glycinamide ribonucleotide transformylase (GART)
1a8i	1a8i_GLS, 1b4d_CRA, 1ftw_GL5, 1k06_BZD, 1ww2_NBG, 1ww3_NTF, 2qn8_NBY	Ref/A: 0.31 ± 0.14 Ref/D: 0.14 ± 0.08 A/A: 0.32 ± 0.16 A/D: 0.13 ± 0.09	Ref/A: 0.62 ± 0.14 Ref/D: 0.51 ± 0.11 A/A: 0.61 ± 0.16 A/D: 0.43 ± 0.13	glycogen phosphorylase b (GPb)
1m2z	3cld_GW6, 1m2z_DEX, 4udd_CV7,	Ref/A: 0.11 ± 0.08 Ref/D: 0.8 ± 0.02 A/A: 0.26 ± 0.21	Ref/A: 0.44 ± 0.13 Ref/D: 0.31 ±	glucocorticoid receptor (GR)

	4p6w_MOF	A/D: 0.09 ± 0.03	0.07<br>A/A: 0.59 ± 0.21<br>A/D: 0.36 ± 0.09	
1hpx	1ajx_AH1, 1g35_AHF, 1hbv_GAN, 1npv_L27, 1ohr_1UN, 1upj_U01, 1xl5_190, 1yt9_OIS, 4hla_017, 7upj_INU, 2qnq_QN3	Ref/A: 0.16 ± 0.10<br>Ref/D: 0.12 ± 0.03<br>A/A: 0.25 ± 0.19<br>A/D: 0.13 ± 0.04	Ref/A: 0.48 ± 0.13<br>Ref/D: 0.47 ± 0.10<br>A/A: 0.52 ± 0.18<br>A/D: 0.49 ± 0.11	HIV-1 protease (HIVPR)
1rt1	1rt1_MKC, 3dya_PZL, 4ncg_2KW, 3ffi_30B, 1c0t_BM1, 1dtt_FTC, 1tkt_H12, 1rti_HEF	Ref/A: 0.12 ± 0.10<br>Ref/D: 0.10 ± 0.03<br>A/A: 0.12 ± 0.10<br>A/D: 0.11 ± 0.04	Ref/A: 0.43 ± 0.12<br>Ref/D: 0.43 ± 0.08<br>A/A: 0.39 ± 0.13<br>A/D: 0.40 ± 0.09	HIV-1 reverse transcriptase (HIVRT)
1hw8	1hw8_114, 1hwi_115, 1hwj_116, 3cct_3HI, 3cd0_6HI, 2q6b_HR2	Ref/A: 0.18 ± 0.14<br>Ref/D: 0.11 ± 0.02<br>A/A: 0.38 ± 0.25<br>A/D: 0.10 ± 0.02	Ref/A: 0.48 ± 0.17<br>Ref/D: 0.45 ± 0.10<br>A/A: 0.70 ± 0.22<br>A/D: 0.36 ± 0.08	HMG-CoA reductase (HMGR)
1uy6	2yi0_Yi0, 2yi7_BZ8, 3b27_B2T, 3bm9_BXZ,	Ref/A: 0.20 ± 0.14<br>Ref/D: 0.10 ± 0.04<br>A/A: 0.25 ± 0.20	Ref/A: 0.51 ± 0.14<br>Ref/D: 0.40 ±	heat shock protein 90

	3eko_PYU, 3o0i_P54, 4lwe_FJ2, 4lwg_FJ4	A/D: 0.10 ± 0.04	0.09<br>A/A: 0.59 ± 0.17<br>A/D: 0.46 ± 0.08	(HSP90)
1p44	1p44_GEQ, 3fne_8PC, 4cod_KV1, 4d0s_9G4, 4r9s_3KY, 4tzk_641, 4u0j_566, 4u0k_744, 4uvi_KXU	Ref/A: 0.13 ± 0.11<br>Ref/D: 0.12 ± 0.04<br>A/A: 0.22 ± 0.20<br>A/D: 0.11 ± 0.04	Ref/A: 0.46 ± 0.14<br>Ref/D: 0.46 ± 0.10<br>A/A: 0.51 ± 0.19<br>A/D: 0.44 ± 0.09	enoyl reductase (InhA)
2aa2	2a3i_C0R, 2aa2_AS4, 2aa5_STR, 2aa7_1CA	Ref/A: 0.32 ± 0.14<br>Ref/D: 0.13 ± 0.07<br>A/A: 0.24 ± 0.19<br>A/D: 0.12 ± 0.06	Ref/A: 0.71 ± 0.17<br>Ref/D: 0.40 ± 0.15<br>A/A: 0.62 ± 0.19<br>A/D: 0.39 ± 0.14	mineralcorticoid receptor (MR)
1a4g	1a4g_ZMR, 1a4q_DPC, 1nsc_SIA, 1nsd_DAN	Ref/A: 0.20 ± 0.07<br>Ref/D: 0.11 ± 0.04<br>A/A: 0.36 ± 0.21<br>A/D: 0.12 ± 0.05	Ref/A: 0.55 ± 0.08<br>Ref/D: 0.45 ± 0.09<br>A/A: 0.64 ± 0.16<br>A/D: 0.43 ± 0.08	neuraminidase (NA)
1wbv	1w82_L10, 1wbv_LI3, 2yis_YIS, 3hv6_R39, 3nnw_EDD	Ref/A: 0.09 ± 0.06<br>Ref/D: 0.11 ± 0.04<br>A/A: 0.19 ± 0.12<br>A/D: 0.11 ± 0.05	Ref/A: 0.46 ± 0.10<br>Ref/D: 0.45 ± 0.09	P38 MAP kinase (P38)

			A/A: 0.50 ± 0.11<br>A/D: 0.45 ± 0.09	
1efy	1efy_BZC, 1pax_DHQ, 2pax_4AN, 3pax_3MB, 4pax_NU1	Ref/A: 0.20 ± 0.09<br>Ref/D: 0.14 ± 0.05<br>A/A: 0.42 ± 0.17<br>A/D: 0.12 ± 0.04	Ref/A: 0.45 ± 0.10<br>Ref/D: 0.43 ± 0.11<br>A/A: 0.74 ± 0.15<br>A/D: 0.45 ± 0.09	poly (ADP-ribose) polymerase (PARP)
1xp0	1tbf_VIA, 1xoz_CIA, 1xp0_VDN	Ref/A: 0.18 ± 0.12<br>Ref/D: 0.12 ± 0.04<br>A/A: 0.13 ± 0.09<br>A/D: 0.11 ± 0.04	Ref/A: 0.61 ± 0.15<br>Ref/D: 0.58 ± 0.09<br>A/A: 0.55 ± 0.11<br>A/D: 0.50 ± 0.11	phoshodiesterase 5a (PDE5)
1b8o	1b8n_IMG, 1b8o_IMH, 2ai1_P1G, 2qpl_BTY, 3fuc_9DG	Ref/A: 0.21 ± 0.10<br>Ref/D: 0.10 ± 0.04<br>A/A: 0.31 ± 0.21<br>A/D: 0.11 ± 0.04	Ref/A: 0.63 ± 0.08<br>Ref/D: 0.50 ± 0.08<br>A/A: 0.71 ± 0.14<br>A/D: 050 ± 0.10	purine nucleoside phosphorylase (PNP)
4yt1	2pob_GW4, 4jl4_AXY, 1k74_544	Ref/A: 0.29 ± 0.10<br>Ref/D: 0.16 ± 0.04<br>A/A: 0.47 ± 0.18<br>A/D: 0.16 ± 0.05	Ref/A: 0.48 ± 0.18<br>Ref/D: 0.45 ± 0.13<br>A/A: 0.71 ± 0.15<br>A/D: 0.53 ± 0.09	peroxisome proliferator activated receptor γ (PPARγ)

1sr7	1a28_STR, 1sqn_NDR, 1sr7_MOF, 1zuc_T98, 3d90_NOG	Ref/A: 0.14 ± 0.12<br>Ref/D: 0.08 ± 0.03<br>A/A: 0.29 ± 0.23<br>A/D: 0.09 ± 0.03	Ref/A: 0.41 ± 0.19<br>Ref/D: 0.31 ± 0.11<br>A/A: 0.58 ± 0.26<br>A/D: 0.37 ± 0.09	progesterone receptor (PR)
1mvc	1mvc_BM6, 2p1t_3TN, 3fug_2E3, 4rmd_3SW	Ref/A: 0.27 ± 0.03<br>Ref/D: 0.12 ± 0.05<br>A/A: 0.54 ± 0.17<br>A/D: 0.16 ± 0.05	Ref/A: 0.52 ± 0.12<br>Ref/D: 0.39 ± 0.10<br>A/A: 0.69 ± 0.13<br>A/D: 0.40 ± 0.08	retinoid X receptor
1a7a	1a7a_ADC	Ref/A: 0.43 ± 0.14<br>Ref/D: 0.10 ± 0.08<br>A/A: 0.37 ± 0.17<br>A/D: 0.11 ± 0.07	Ref/A: 0.80 ± 0.08<br>Ref/D: 0.50 ± 0.10<br>A/A: 0.78 ± 0.09<br>A/D: 0.51 ± 0.11	S-adenosyl-homocysteine hydrolase (SAHH)
1y57	1y57_MPZ	Ref/A: 0.15 ± 0.05<br>Ref/D: 0.08 ± 0.02<br>A/A: 0.22 ± 0.19<br>A/D: 0.11 ± 0.04	Ref/A: 0.53 ± 0.12<br>Ref/D: 0.45 ± 0.07<br>A/A: 0.57 ± 0.16<br>A/D: 0.46 ± 0.09	cellular sarcoma tyrosine kinase (c-SRC)
1ba8	1a4w_QWE,	Ref/A: 0.13 ± 0.07	Ref/A: 0.47 ±	thrombin

	1c5n_ESI, 1o2g_696, 4lxb_7R9	Ref/D: 0.14 ± 0.03 A/A: 0.22 ± 0.19 A/D: 0.13 ± 0.05	0.21 Ref/D: 0.43 ± 0.16 A/A: 0.57 ± 0.16 A/D: 0.51 ± 0.12	
1kim	1e2i_APS, 1e2i_ARP, 1e2k_TMC, 1qhi_BPG, 4ivq_I43, 4jbx_SK7	Ref/A: 0.15 ± 0.09 Ref/D: 0.11 ± 0.06 A/A: 0.32 ± 0.19 A/D: 0.13 ± 0.09	Ref/A: 0.59 ± 0.14 Ref/D: 0.47 ± 0.12 A/A: 0.74 ± 0.14 A/D: 0.52 ± 0.15	thymidine kinase (TK)
1bju	1c1q_BAI, 1gi0_BMZ, 1zzz_0IV, 1pph_0ZG, 1k1i_FD1	Ref/A: 0.24 ± 0.20 Ref/D: 0.12 ± 0.04 A/A: 0.35 ± 0.25 A/D: 0.13 ± 0.05	Ref/A: 0.57 ± 0.25 Ref/D: 0.39 ± 0.08 A/A: 0.65 ± 0.24 A/D: 0.52 ± 0.12	trypsin
4ag8	2xir_00J, 3wzd_LEV, 3wze_BAX, 4ag8_AXI, 5ew3_5T2	Ref/A: 0.13 ± 0.07 Ref/D: 0.13 ± 0.04 A/A: 0.13 ± 0.10 A/D: 0.11 ± 0.04	Ref/A: 0.46 ± 0.10 Ref/D: 0.48 ± 0.08 A/A: 0.46 ± 0.11 A/D: 0.44 ± 0.09	vascular endothelial growth factor receptor 2 (VEGFR2)

S4 - Analysis of dataset diversity

For all ligand datasets, the pairwise ECFP4 [1] Tanimoto similarities were calculated using PipelinePilot [2].

	< 0.1	< 0.2	< 0.3	< 0.4	< 0.5	< 0.6	< 0.7	< 0.8	< 0.9	< 1	1
**Nuclear Hormone Receptors**											
**AR**	24.5	41.2	14.3	6.7	4.2	5.9	2.0	0.7	0.1	0.0	0.3
**ER (ag.)**	20.7	41.8	16.1	8.1	5.4	3.9	2.1	1.1	0.3	0.0	0.4
**ER (ant.)**	3.4	49.9	12.4	11.1	9.2	6.1	3.8	2.8	1.3	0.0	0.0
**GR**	39.1	13.3	7.0	10.3	14.9	6.5	4.3	3.7	0.8	0.0	0.1
**MR**	17.1	23.8	24.8	15.2	6.7	6.7	2.9	1.0	0.0	0.0	1.9
**PPARg**	0.9	12.3	5.5	10.2	27.8	19.3	12.5	9.1	2.3	0.2	0.0
**PR**	43.0	2.0	19.7	2.0	6.3	16.2	9.4	1.1	0.3	0.0	0.0
**RXRa**	0.0	0.0	8.9	17.9	8.4	27.4	15.3	18.9	3.2	0.0	0.0
**Kinases**											
**CDK2**	57.6	29.8	5.6	2.5	2.0	1.1	0.9	0.6	0.0	0.0	0.0
**EGFR**	3.0	27.3	27.7	20.1	14.1	5.2	1.8	0.6	0.2	0.0	0.0
**FGFR1**	26.8	25.5	12.1	12.2	8.1	6.2	5.6	2.7	0.6	0.2	0.0
**HSP90**	22.8	31.9	8.7	6.9	13.0	4.0	5.8	5.8	1.1	0.0	0.0
**P38**	22.6	25.7	26.6	11.3	6.7	3.8	2.3	0.7	0.1	0.0	0.1
**SRC**	35.6	28.2	7.5	9.1	7.0	5.5	4.5	2.1	0.4	0.1	0.0
**TK**	0.9	42.9	8.2	16.9	13.9	5.6	8.7	3.0	0.0	0.0	0.0
**VEGFR2**	45.4	43.5	5.8	2.1	1.3	0.8	0.4	0.6	0.0	0.0	0.0
**Serine Proteases**											
**Fxa**	8.1	20.0	23.8	9.7	14.5	11.7	7.7	3.3	1.0	0.1	0.0
**thrombin**	17.9	26.1	6.5	3.4	10.5	14.8	14.2	4.2	2.1	0.2	0.1
**trypsin**	14.2	24.4	2.9	4.0	15.9	15.3	13.1	7.2	2.4	0.3	0.3
**Metalloenzymes**											
**ACE**	0.9	33.3	39.0	13.4	6.5	2.7	0.8	2.1	0.1	0.0	1.2
**ADA**	27.7	25.7	10.7	3.6	22.9	4.7	4.7	0.0	0.0	0.0	0.0
**COMT**	14.5	30.9	25.5	1.8	14.5	1.8	0.0	5.5	3.6	0.0	1.8
**PDE5**	40.5	44.4	6.8	4.5	1.5	1.1	0.9	0.4	0.0	0.0	0.0
**Folate Enzymes**											
**DHFR**	0.0	27.2	39.2	17.2	9.6	4.1	1.8	0.8	0.1	0.0	0.0
**GART**	0.0	0.0	0.0	21.0	38.1	16.7	10.5	10.0	1.9	0.5	1.4
**Other Enzymes**											
**AChE**	21.0	17.1	10.8	22.5	14.3	7.0	4.4	2.5	0.5	0.0	0.0
**ALR2**	35.4	48.0	5.2	2.2	2.8	1.8	1.8	0.3	0.6	0.0	1.8
**AmpC**	0.0	20.0	18.1	15.7	17.1	16.2	7.1	5.2	0.5	0.0	0.0
**COX-1**	13.3	54.7	22.0	6.0	1.3	0.3	0.0	1.3	0.3	0.0	0.7
**COX-2**	12.3	33.4	33.5	12.7	4.0	2.2	1.3	0.4	0.1	0.0	0.0
**GPB**	9.0	9.3	32.7	21.3	15.0	6.7	4.4	1.3	0.0	0.0	0.4
**HIVPR**	10.2	48.1	9.8	8.1	8.2	6.4	4.4	3.2	1.5	0.0	0.1
**HIVRT**	56.2	35.0	3.1	2.1	0.4	1.2	1.2	0.4	0.1	0.0	0.5
**HMGR**	23.5	6.4	16.0	9.6	14.3	11.4	7.6	7.2	2.4	0.0	1.7
**InhA**	36.5	28.6	3.0	6.1	10.6	6.2	5.6	2.8	0.4	0.0	0.0
**NA**	0.8	33.1	20.9	8.4	10.2	10.5	10.5	2.6	1.2	0.9	1.0
**PARP**	1.9	13.1	8.3	35.0	15.5	11.4	9.7	4.4	0.8	0.0	0.0
**PNP**	11.7	24.7	13.0	18.7	13.0	7.7	4.7	6.3	0.3	0.0	0.0
**SAHH**	2.8	8.3	27.7	16.9	24.1	11.4	4.0	3.2	0.4	0.0	1.3

Figure S2: Percentage of pairwise ECFP4 Tanimoto similarities for the different protein datasets assigned to bins of increment 0.1.

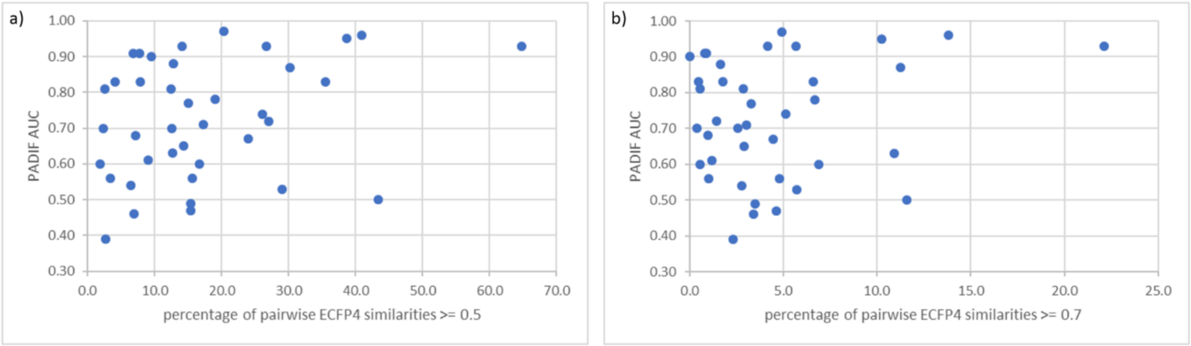

Figure S3: Plot of the PADIF AUC values versus the percentage of pairwise ligand ECFP4 similarities (a) >= 0.5 and (b) >= 0.7 for the different protein datasets.

Bibliography

1. Rogers D, Hahn M. Extended-connectivity fingerprints. J Chem Inf Model. 2010;50:742–54.

2. Pipeline Pilot. Dassault Systèmes BIOVIA, Discovery Studio Modeling Environment, Release 2016, San Diego. 2016.
